# Tankyrase inhibition interferes with junction remodeling, induces leakiness, and disturbs YAP1/TAZ signaling in the endothelium

**DOI:** 10.1007/s00210-023-02720-1

**Published:** 2023-09-23

**Authors:** Nan Ma, Yohanes Cakrapradipta Wibowo, Phillip Wirtz, Doris Baltus, Thomas Wieland, Sepp Jansen

**Affiliations:** 1 Experimental Pharmacology Mannheim, European Center for Angioscience (ECAS), Mannheim Medical Faculty, Heidelberg University, Mannheim, Germany; 2 DZHK, German Center for Cardiovascular Research, partner site Heidelberg/Mannheim, Mannheim, Germany

**Keywords:** Endothelium, Tankyrase, YAP1, TAZ, Actin cytoskeleton, XAV939

## Abstract

Tankyrase inhibitors are increasingly considered for therapeutic use in malignancies that are characterized by high intrinsic β-catenin activity. However, how tankyrase inhibition affects the endothelium after systemic application remains poorly understood. In this study, we aimed to investigate how the tankyrase inhibitor XAV939 affects endothelial cell function and the underlying mechanism involved. Endothelial cell function was analyzed using sprouting angiogenesis, endothelial cell migration, junctional dynamics, and permeability using human umbilical vein endothelial cells (HUVEC) and explanted mouse retina. Underlying signaling was studied using western blot, immunofluorescence, and qPCR in HUVEC in addition to luciferase reporter gene assays in human embryonic kidney cells. XAV939 treatment leads to altered junctional dynamics and permeability as well as impaired endothelial migration. Mechanistically, XAV939 increased stability of the angiomotin-like proteins 1 and 2, which impedes the nuclear translocation of YAP1/TAZ and consequently suppresses TEAD-mediated transcription. Intriguingly, XAV939 disrupts adherens junctions by inducing RhoA-Rho dependent kinase (ROCK)-mediated F-actin bundling, whereas disruption of F-actin bundling through the ROCK inhibitor H1152 restores endothelial cell function. Unexpectedly, this was accompanied by an increase in nuclear TAZ and TEAD-mediated transcription, suggesting differential regulation of YAP1 and TAZ by the actin cytoskeleton in endothelial cells. In conclusion, our findings elucidate the complex relationship between the actin cytoskeleton, YAP1/TAZ signaling, and endothelial cell function and how tankyrase inhibition disturbs this well-balanced signaling.

## Introduction

Tankyrases are a family of enzymes belonging to the PARP superfamily which was recently named as adenosine diphosphate (ADP)ribosyltransferases (ARTD). Tankyrases have two isoforms, tankyrase 1 (also known as PARP5a or ARTD5) and tankyrase 2 (PARP5b or ARTD6), which are involved in vital biological functions, such as telomere homeostasis, glucose homeostasis, and ubiquitination of proteins and proteasome function. The increased understanding of the roles tankyrases play in cancer progression has led to the development of an expanding collection of low molecular weight inhibitors suitable for preclinical characterization and more recently to compounds with improved pharmacokinetic properties for potential clinical use, of which three have entered clinical trials namely, stenoparib (Plummer et al. 2020), JPI547 (NCT04335604), and STP06−1002 (NCT04505839) (Kim et al. 2022). Because they interfere with high potency with Wnt/β-catenin signaling, their therapeutic potential is mostly regarded in the context of malignant tumors that exhibit a high intrinsic β-catenin activity, such as colorectal and hepatocellular carcinoma. Indeed, the first tankyrase inhibitors were discovered in phenotypic screens designed to identify novel canonical Wnt/β-catenin antagonists (Huang et al. 2009, Waaler et al. 2011, James et al. 2012, Okada-Iwasaki et al. 2016). As such, especially their role in regulating pro-oncogenic Wnt/β-catenin signaling is best understood (Croy et al. 2016) and has generally received much excitement considering the limited number of targetable enzymes in the pathway. Among the identified tankyrase inhibitors, XAV939 (Huang et al. 2009) has been the most widely studied and exhibits potent efficacy and antitumor activity in in vitro and in vivo preclinical tumor models (Luo et al. 2022, Neiheisel et al. 2022).

The Wnt/β‐catenin signaling pathway plays a major role in development, tissue homeostasis, and pathology (Nusse and Clevers 2017, Zhan et al. 2017). The central intracellular signaling protein is β-catenin, which exists in different intracellular pools, i.e., a relative majority at adherens junctions (junctional pool) and a relative cytosolic minority that can translocate to the nucleus where it functions as a transcriptional co-factor (cytosolic pool). Central to the pathway is a multiprotein complex that regulates the stability of the cytosolic pool by phosphorylation, ubiquitination, and subsequent proteasomal degradation. Importantly, the establishment of this complex is believed to be dependent on the availability of the concentration limiting component axin (axin1/axin2, the form is redundant in Wnt/β-catenin signaling); therefore, controlling axin is an effective way to regulate the cytosolic pool of β-catenin. Axin is polyADP-ribosylated (PARsylated) by tankyrases, which results in subsequent ubiquitination by the E3 ubiquitin ligase RNF146 and proteasomal degradation of axin (Zhang et al. 2011). As such, tankyrase inhibition increases axin availability and thereby accelerates degradation of the cytosolic pool with minimal effect on the junctional pool. Indeed, tankyrase inhibition in colorectal cancer cells lines gives rise to axin stabilization, reduced cytosolic β-catenin, and attenuated β‐catenin‐dependent transcription (Lau et al. 2013, Mariotti et al. 2017, Neiheisel et al. 2022).

However, important to note is that the Wnt/β-catenin pathway is by no means exclusive to malignant cells, but is ubiquitously expressed and involved in virtually all cell types. Regarding antitumor activity of tankyrase inhibitors, especially its effect on the (tumor) vasculature deserves careful consideration as any systemic application of tankyrase inhibitors would come directly in contact with and pass the endothelial cells (EC) lining the vasculature. Here, canonical Wnt/β-catenin signaling is involved in proliferation and migration of EC, two important aspects during angiogenesis (Masckauchan et al. 2005, Samarzija et al. 2009), vascular remodeling (Corada et al. 2010), and junctional integrity (Tran et al. 2016). Especially during disease states characterized by pathological angiogenesis increased endothelial nuclear β-catenin accumulation is a common hallmark (Manoranjan and Provias 2022), whereas its role junctional integrity makes it an enticing therapeutic target for conditions characterized by pathological angiogenesis that would also profit from transiently opening the endothelial barrier in order to facilitate drug delivery to the surrounding tissue, such as malignant tumors. However, outside of the brain vasculature (Ben-Zvi and Liebner 2022), endothelial β-catenin signaling is relatively poorly understood.

Generally, studies probing the role of endothelial β-catenin employ β-catenin-deficient EC in vitro or postnatal ablation of endothelial β-catenin in vivo; however, such studies struggle in discriminating between junctional and signaling functions of β-catenin as depletion affects both intracellular pools. To specifically evaluate the role of β-catenin as a transcription factor, a different approach is needed that only affects the cytosolic pool, such as increasing axin availability. Indeed, EC overexpression of axin1 in EC has proven successful in inducing retinal hypovascularization (Martowicz et al. 2019), suggesting the involvement of β-catenin-dependent transcription in this process. However, while this is experimentally a valuable tool, this approach has at present no therapeutic application. Here, tankyrase inhibitors provides an enticing pharmacological tool to simultaneously elucidate β-catenin signaling and tankyrase function in EC. To date, only a few studies have demonstrated the efficacy of the tankyrase inhibitor XAV939 in EC in preventing chronic thromboembolic pulmonary hypertension (Zeng et al. 2021), aggravating blood-brain barrier breakdown in ischemic stroke (Jean LeBlanc et al. 2019) and preventing corneal neovascularization (Zhong et al. 2021). However, none of these studies have accurately demonstrated that the effects of XAV939 could be attributed to inhibition of β-catenin signaling alone. In this regard, it is especially important to note that tankyrases have recently been implicated as regulators of other major signaling pathways for EC function, including the Hippo pathway (Wang et al. 2015, Wang et al. 2016a) mediated by its core components Yes-associated protein 1 (YAP1) and its paralogue transcriptional coactivator with PDZ-binding motif (TAZ, also known as WWTR1). Although this pathway is commonly found to be co-regulated with Wnt/β-catenin signaling (Rosenbluh et al. 2012, Azzolin et al. 2014, Benham-Pyle et al. 2015), tankyrases interact with this pathway by regulating the motin family of proteins (angiomotin, angiomotin-like 1 and 2 (AMOTL1, AMOTL2) through promoting PARsylation-dependent degradation (Wang et al. 2015, Troilo et al. 2016, Wang et al. 2016a).

Here, we aimed to study the effect of tankyrase inhibition using XAV939 on EC function with the goal of providing further insights on its role in EC β-catenin and YAP1/TAZ signaling. We will provide evidence that in EC, tankyrase inhibition induces junctional remodeling and alters gene transcription by altering YAP1 and TAZ availability.

## Materials and methods

### Reagents

Seventy-kilodalton fluorescein (FITC)-labeled dextran (#FD70S) and tetramethylrhodamine-5-isothiocyanate (TRITC)-conjugated phalloidin (#P1951) were purchased from Sigma-Aldrich. Recombinant human vascular endothelial growth factor (VEGF) (#293-VE) was from Bio-Techne. 3,5,7,8-Tetrahydro-2-[4-(trifluoromethyl)phenyl]-4H-thiopyrano[4,3-d]pyrimidin-4-one (XAV939, #3748) and (S)-(+)-2-methyl-1-[(4-methyl-5-isoquinolinyl)sulfonyl]-hexahydro-1H-1,4-diazepine dihydrochloride (H1152, #2414) were from Tocris. XAV939 was diluted to a 20 mM working stock solution in dimethyl sulfoxide (DMSO). All other reagents used were of analytical grade.

### Human umbilical vein endothelial cell (HUVEC) isolation and cell culture

HUVECs were isolated from fresh human umbilical cords obtained from University Medical Center Mannheim (UMM) as described previously (Baudin et al. 2007). Cells were seeded on 0.25% gelatin-coated dishes and cultured in EC growth medium (C-22110, PromoCell) containing supplements (C-39210, PromoCell) and 2% (v/v) fetal calf serum (FCS) (C-37350, PromoCell). Cells were used at passages 2 to 4 for experiments. Human embryonic kidney (HEK-293) cells were obtained from the American Type Culture Collection (#CRL-1573, ATCC) and grown in Dulbecco’s modified Eagle’s medium (DMEM) supplemented with 10% FCS, 4 mM L-glutamine, and 1% penicillin/streptomycin. Cells were kept under standard cell culture conditions (37 °C, 5% carbon dioxide (CO_2_)). For oscillatory shear stress, HUVECs (75,000 per channel) were seeded onto µ-Slide VI 0.4 (IBIDI). µ-Slides were installed into an IBIDI Pump System set up for oscillatory shear stress (12 dyne/cm^2^ at 2 Hz). After 24 h, cells were fixed with 3.7% paraformaldehyde (PFA) in Hank’s balanced salt solution (HBSS) and prepared for immunofluorescence (IF).

### Small interfering RNA (siRNA) transfection

Cells were transfected with either control siRNA (1027281, Qiagen) or siRNA targeting β-catenin (siCTNNB1, ON-TARGETplus human CTNNB1, Dharmacom), YAP1 (siYAP1, ON-TARGETplus human YAP1, Dharmacom), and Taz (siTaz, ON-TARGETplus human WWTR1, Dharmacom) at 60–70% confluency in serum- and antibiotics-free Opti-MEM™ medium (Gibco). Cells were transfected in 6-well plates using 250 pmol siRNA per well. Transfection was performed according to the manufacturer’s protocol (Lipofectamine® RNAiMAX Transfection Reagent, Invitrogen).

### Plasmid transfection

Cells were transfected with plasmid DNA at 60–70% confluency in serum- and antibiotics-free medium. The following plasmids were used: TOPFlash (Veeman et al. 2003), FOPFlash (Veeman et al. 2003), HOPFlash (Kim and Gumbiner 2015), HIPFlash (Kim and Gumbiner 2015), FHRE-Luc (Brunet et al. 1999), β-catenin^S33Y^ (Kolligs et al. 1999), YAP1-eGFP (Basu et al. 2003), and Gα_13_Q226L. Gα_13_Q226L was from cDNA Resource Center (cdna.org), all other plasmids were from Addgene (addgene.org) and we kindly thank the original depositors for kindly making them available. Transfection was performed using PolyFect (Qiagen) according to the manufacturer’s protocol.

### Lentivirus generation and transduction

pLL3.7-EGFPC2-TAZ (Bao et al. 2011) and pLenti Lifeact-mRuby2 (Parker et al. 2018) were packed into a 3rd-generation lentiviral packaging system (pMD2.G, pMDLg/pRRE, pRSV-Rev (Dull et al. 1998)) using HEK293T cells (CRL-3216, ATCC). All plasmids were from Addgene (addgene.org), and we kindly thank the original depositors for kindly making them available. Transfection was performed using X-tremeGENE™ 9 DNA Transfection Reagent (Roche) according to the manufacturer’s recommendations. 10 mM sodium butyrate was added to improve viral vector production. Supernatant was collected at 24 h, 48 h, and 72 h, and viral particles were collected using Vivaspin® 6 columns (Sartorius). Concentrated virus was stored in single-use aliquots at −80 °C until further use. For viral transduction, 50,000 HUVECs were seeded per well in a 12-well plate. Lentiviral transduction was done for 48 h in the presence of 4 µg/ml polybrene at an MOI of 100. Subsequently, cells were puromycin selected at 2 µg/ml for 48 h. Transduced HUVECs were then expanded and prepared for downstream use in experiments.

### Ex vivo retinal angiogenesis

Mouse retinas from post-natal day 5 (p5) eyes were dissected and flat-mounted on Millicell Cell Culture Inserts (Millipore) as described previously (Sawamiphak et al. 2010). Briefly, retinas were cultured in DMEM with 10% FCS at 35 °C in a humidified incubator (5% CO_2_) until attached to the membrane (2–4 h). XAV939 was diluted in 3% FCS DMEM to a final concentration of 5 μM. Retinal explants were then subjected to XAV939 treatment (16 h) at 35 °C in a humidified incubator (5% CO_2_). Retinas were fixed in 4% PFA at 35 °C for 4 h, blocked with 3% bovine serum albumin (BSA), and permeabilized with 0.1% Triton X-100 (both in CytoTBS-T buffer (20 mM Tris hydrochloride (Tris-HCl), 154 mM sodium chloride (NaCl), 2 mM ethylene glycol tetraacetic acid (EGTA), 2 mM magnesium chloride (MgCl_2_), 0.1% v/v Tween 20, pH 7.2)) and stained with FITC-conjugated isolectin-B4 (1:200, L9381, Sigma-Aldrich) overnight at 4 °C. To visualize the retinal vasculature, images were acquired with a microscope (Zeiss, LSM700). Animal care and experiments were performed in accordance with the Association for Research in Vision and Ophthalmology (ARVO) statement and were approved by the local government (I-18/21 (“Interne Anzeige,” Regierungspräsidium Karlsruhe, Germany). All efforts were made to reduce the animal number as well as the animal suffering.

### Monomeric GTPase activity assay

The cellular levels of active RhoA and CDC42 were determined using effector pulldown assays as described previously (Lutz et al. 2004). Briefly, serum-starved HUVEC were stimulated with XAV939 after which cells were lysed in ice-cold GST-FISH buffer (10% glycerol, 50 mM Tris-HCl, pH 7.4, 100 mM NaCl, 1% NP-40, 2 mM MgCl_2_). The guanosine triphosphate (GTP)-bound monomeric GTPases were precipitated with either the CDC42 (p21)-binding domain (PBD) of p21 activated kinase 1 (PAK1) or the Rho binding domain (RBD) of rhotekin coupled to glutathione sepharose. The amounts of activated (GTP-bound) and total GTPases were then determined by western blot analysis.

### Western blot analysis

Cells were lysed in RIPA buffer supplemented with cOmplete™ Protease Inhibitor Cocktail and PhosSTOP™ phosphatase inhibitors (Roche Applied Sciences), and proteins were separated in denaturing acrylamide gels (6–12% acrylamide) and subsequently transferred to nitrocellulose membranes (GE10600000, Amersham). After blocking with Roti® Block (Carl Roth) for 1 h, the membranes were probed with primary antibodies overnight at 4 °C followed by incubation with appropriate HRP-conjugated secondary antibodies. The immuno-reactive bands were visualized with enhanced chemiluminescence (34096, Thermo Fischer Scientific) and then quantified by the densitometry analysis using ImageJ. The following primary antibodies were used for western blots: β-catenin (8480, Cell Signaling), axin1 (3323, Cell Signaling), GAPDH (sc-47724, Santa Cruz), VE-cadherin (2500, Cell Signaling), claudin (35-2500, Invitrogen), AMOTL1 (HPA001196, Sigma-Aldrich), AMOTL2 (23351-1-AP, Proteintech), angiomotin (43130, Cell Signaling), p190RhoGAP (610150, BD Biosciences), IQGAP1 (sc-10792, Santa Cruz), YAP1 (PA1-46189, Thermo Fischer Scientific), TAZ (560235, BD Biosciences), VEGFR2 (2479, Cell Signaling), CD31 (551262, BD Biosciences), tubulin (T6557, Sigma-Aldrich), p-VEGFR2^Y1175^ (2478, Cell Signaling), p-Akt^S473^ (9271, Cell Signaling), p-ERK^S42/S44^ (4370, Cell Signaling), p-AKT-substrate (9614, Cell Signaling), CDC42 (2462, Cell Signaling), vinculin (13901, Cell Signaling), HRP-conjugated anti-mouse (A9044, Sigma-Aldrich), and HRP-conjugated anti-rabbit (A9169, Sigma-Aldrich).

### Isolation of mRNA and real-time PCR

Cells were lysed in RLT Buffer (Qiagen), and total RNA was isolated according to the manufacturer’s recommendations (RNeasy Mini Kit, 74104, Qiagen) followed by cDNA synthesis (GoScript™ Reverse Transcriptase cDNA synthesis kit, Promega). Multiplex real-time PCR was performed in triplicates by using master mix (KK4705, Kapa Biosystems) and the TaqMan probes for either ANKDR1 (Hs00998537, Invitrogen), VEGFR2 (Hs00911700, Invitrogen), CTNNB1 (Hs00170025, Invitrogen), CCND1 (Hs00765553, Invitrogen), MYC (Hs00153408, Invitrogen), CTGF (Hs00170014, Invitrogen), and CYR61 (Hs00205294, Invitrogen) while B2M (Hs00187842, Invitrogen) and HPRT (Hs02800695, Invitrogen) were used as internal controls. A program of 50 °C × 2 min and 95 °C × 10 min followed by 40 cycles of 95 °C × 15 s and 60 °C × 1 min was performed on Applied Biosystems’ StepOnePlus Real-Time PCR thermocycler in a 96-well plate format. Data were analyzed using the ΔΔCt method to determine the relative expression.

### Sprouting assay

In vitro sprouting angiogenesis assays with HUVECs were performed as described previously (Heiss et al. 2015). Briefly, 400 cells were mixed with methyl cellulose and spotted dropwise on a culture dish, which was then incubated upside down for 24 h to generate the cellular spheroids. Spheroids were then embedded into a 3D collagen-based gel matrix pre-prepared from rat tail tendons followed by stimulation with the indicated reagents for 24 h. The cumulative sprout length was measured by microscopy (Olympus IX81) using Olympus cellSens software. In an individual experiment, around 10 spheroids were analyzed for each condition. In experiments involving siRNAs, the cells were transfected with the indicated siRNAs, 1 day prior to the generation of the spheroids.

### Cell migration assay

Migration assay was performed by using Oris™ Cell Migration Assay (CMA5.101, Platypus Technologies). After insertion of Oris™ Cell Seeding Stoppers, 50,000 HUVECs were seeded in the Oris™ 96-well plate. Cell migration started by removal of the Oris™ Cell Seeding Stoppers and was performed in the presence of 1 µg/ml aphidicolin (5736, Tocris). Migration was stopped after 24 h, and cells were stained with 0.1 µM Calcein AM cell-permeant fluorescent dye (354217, Corning) for 30 min. The Oris™ Detection Mask was applied to the bottom of the plate to allow for measurement of the fluorescent signal using a multi-label plate reader (Envision 2102, Perkin Elmer) by measuring the fluorescence intensity (Ex/Em: 485/535 nm) of the area blocked by the Oris™ Cell Seeding Stoppers during cell seeding.

### Permeability assay

Endothelial permeability was assayed by monitoring the passage of FITC-labeled 70-kDa dextran through a monolayer of HUVEC grown to confluence. Briefly, HUVECs were seeded on Transwell cell culture inserts with a pore size of 3 μm (3462, Corning) until fully confluent and then serum starved overnight and pre-incubated with XAV939 and H1152. After pre-incubation, 2 μM FITC-dextran was added to the upper Transwell compartment. A sample from the bottom compartment was taken every 10 min to measure passage of FITC-dextran through the monolayer using a multi-label plate reader (Envision 2102, Perkin Elmer) by measuring the fluorescence intensity (Ex/Em: 485/535 nm).

### Luciferase reporter gene assays

For luciferase reporter gene assays, cells were transfected with luciferase reporter plasmids (TOPFlash, FOPFlash, HOPFlash, HIPFlash, FHRE-Luc) 24 h prior to addition of indicated substances. An additional Renilla normalization plasmid was included during each transfection (pRL-TK vector, Promega). In case of additional siRNA transfection, the siRNA transfection was done an additional 24 h before luciferase reporter transfection. Treatment with indicated substances was 24h after which cells were lysed and firefly and Renilla luciferase activity was assayed in a multi-label plate reader (Envision 2102, Perkin Elmer) using the Dual Reporter Luciferase Assay System (E1960, Promega) according to the manufacturer’s recommendations.

### Co-immunoprecipitation

Cells were lysed in non-denaturing ice-cold lysis buffer (20 mM Tris-HCl, 150 mM NaCl, 1 mM ethylenediaminetetraacetic acid (EDTA), 1 mM EGTA, 1% (v/v) Triton X-100, pH 7.6) supplemented with cOmplete™ Protease Inhibitor Cocktail and PhosSTOP™ phosphatase inhibitors (Roche Applied Science). Lysates were cleared for 10 min at 14,000×g at 4 °C. Subsequently, 500 μg protein was incubated with 1 μg primary antibody. The following antibodies were used: β-Catenin (8480, Cell Signaling), VE-cadherin (2500, Cell Signaling), AMOTL1 (HPA001196, Sigma-Aldrich), AMOTL2 (23351-1-AP, Proteintech), YAP1 (PA1-46189, Thermo Fischer Scientific), and TAZ (560235, BD Biosciences) overnight at 4 °C with gentle rocking. Subsequently, protein A/G magnetic beads (88803, Thermo Fisher) were added for an additional 2 h. Beads were collected using magnetism and extensively washed with lysis buffer. Beads were then heated for 5 min at 95 °C in denaturing sodium dodecyl sulfate buffer before western blot analysis.

### Immunofluorescence

Cells were grown on Millicell EZ Slides (PEZGS0816, Merck), fixed with PFA, and permeabilized with 0.3% v/v Triton X-100 in cytoskeletal buffer (10 mM Tris-HCl, 150 mM NaCl, 5 mM EGTA, 5 mM MgCl_2_, 5 mM glucose, pH 7.1). Cells were blocked using 1% w/v BSA and 2% v/v goat serum in CytoTBS-T (20 mM Tris-HCl, 154 mM NaCl, 2 mM EGTA, 2 mM MgCl_2_, 0.1% v/v Tween-20, pH 7.2). Primary antibodies were applied overnight at 4 °C with gentle rocking. The following antibodies were used: β-Catenin (8480, Cell Signaling), VE-cadherin (2500, Cell Signaling), AMOTL1 (HPA001196, Sigma-Aldrich), AMOTL2 (23351-1-AP, Proteintech), VEGFR2 (MA5-15157, Thermo Fischer Scientific), TGN38 (sc-166594, Santa Cruz), vinculin (13901, Cell Signaling), YAP1 (PA1-46189, Thermo Fischer Scientific), and TAZ (560235, BD Biosciences). For some experiments, cells were already expressing a green fluorescent protein (GFP)- or red fluorescent mRuby2-tagged construct (YAP1-GFP, TAZ-GFP, LifeAct-mRuby2). Species-appropriate Alexa Fluor 488 or Alexa 594 conjugated secondary antibodies (Life Technologies) were applied for 1 h at RT. For analysis of the actin cytoskeleton, TRITC-conjugated phalloidin was used. Nucleus was visualized with 4′,6-diamidino-2-phenylindole (DAPI) (Sigma). Slides were mounted with Roti-Mount FluorCare (HP19.1, Carl Roth). Images were captured as z-stacks consisting of 10 optical slices of 1 µm using a confocal microscope (Zeiss, LSM700) and analyzed using ImageJ software. Cell junction morphology analysis was done in confluent monolayers of HUVEC stained for vascular endothelial (VE)-Cadherin, as previously described (Neto et al. 2018). Five morphological categories were defined: straight, thick, thick to reticular, reticular, and fingers. The retinal VE-cadherin phenotype was analyzed as previously described (Bentley et al. 2014). Patches were classified into six classes from highly active to highly inhibited.

### Cell surface biotinylation assay

Extracellular proteins were biotinylated by 1-h incubation with sulfo-NHS-LC-biotin (ab145611, Abcam) for 1 h. After quenching with 50 mM glycine, cells were lysed in lysis buffer (50 mM Tris-HCl, 150 mM NaCl, 1 mM EDTA, 1% (v/v) Triton X-100, pH 7.4)) supplemented with cOmplete™ Protease Inhibitor Cocktail (Roche Applied Sciences) for 1 h at 4 °C in a rotating wheel. The biotinylated proteins were pulled down by overnight incubating with Immobilized/NeutrAvidin Ultralink Resin beads (53150, Thermo Fisher Scientific) at 4 °C in a rotating wheel. After extensive washing, the beads were resuspended in denaturing sodium dodecyl sulfate buffer and heated to 70 °C for 10 min before western blot analysis.

### Statistics

The statistical analysis was performed using GraphPad Prism 7. All results are given as mean ± SEM. To test for normal distribution, the *F*-test was used. Data of two groups were compared using the Student’s *t*-test. The analysis and comparison of three and more groups were performed with (ANOVA) and Bonferroni post-hoc test. *p*-values ≤0.05 were considered statistically significant.

## Results

### XAV939 does not affect β-catenin transcriptional activity in EC

**Fig. 1:**
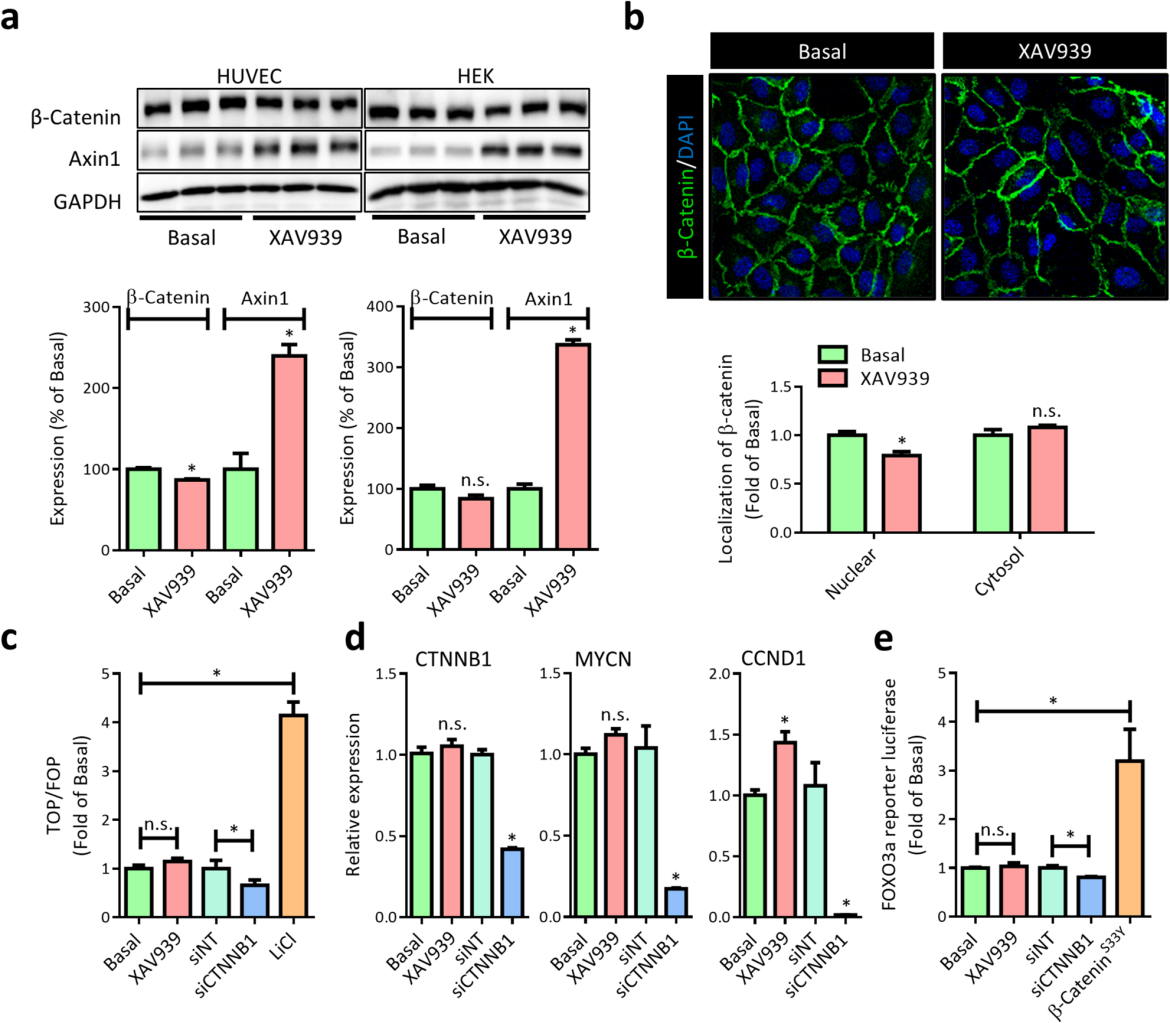
XAV939 treatment does not affect β-catenin transcriptional activity in EC. **a** Representative Western blot (WB) images and quantification of β-catenin and axin1 protein levels in HUVEC and HEK cells after XAV939 (5 µM, 6h) treatment. Data are normalized to Basal and represent mean ± SEM of 3 independent experiments. **b** Representative confocal microscopy images of β-catenin (green) IF and quantification of cytosolic and nuclear β-catenin signal intensity in HUVEC after XAV939 treatment (5 µM, 6h). Nuclei were counterstained with DAPI (blue). Data are normalized to Basal and represent mean ± SEM of 4 independent experiments. **c** TCF/Lef luciferase reporter gene assay (TOPFlash) in HEK cells treated with XAV939 (5 µM, 24 h), and β-catenin knockdown (siCTNNB1). The GSK3β inhibitor LiCl (20 mM, 24 h) acted as a positive control. Data are normalized for non-specific reporter gene activity (FOPFlash) and to the respective control (Basal or non-targeting control siRNA (siNT)) and represent mean ± SEM of 4 independent measurements. **d** qPCR for β-catenin gene expression (CTNNB1) and the TCF/Lef target genes n-Myc (MYCN) and Cyclin D1 (CCND1) in HUVEC treated with XAV939 (5 µM, 6 h), β-catenin knockdown (siCTNNB1). Data are normalized to the respective control (Basal or non-targeting control siRNA (siNT)) and represent mean ± SEM of 3 independent experiments. **e** FOXO3a reporter gene assay in HEK cells treated with XAV939 (5 µM, 24 h), β-catenin knockdown (siCTNNB1). Overexpression of degradation-resistant β-catenin^S33Y^ was used as a positive control. Data are normalized to the respective control (Basal or non-targeting control siRNA (siNT)) and represent mean ± SEM of 4 independent measurements. *p < 0.05, n.s. *p* > 0.05 versus respective control condition or between conditions as indicated by brackets

To study the effect of tankyrase inhibitors on β-catenin in EC, the well-characterized inhibitor XAV939 was used (Huang et al. 2009). As expected, HUVECs and HEK cells treated with XAV939 (6 h) showed a marked increase in axin1 protein levels (Fig. 1a). As axin1 is the concentration-limiting component of the multiprotein complex responsible for phosphorylation and ubiquitination of β-catenin, a reduction in β-catenin protein levels was anticipated. However, results were mixed; in HUVECs, there was a small but significant decrease whereas in HEK cells levels were unaffected. Since the majority of β-catenin in EC resides at cell-cell junctions bound to vascular endothelial (VE)-cadherin and is inaccessible to phosphorylation by axin-associated kinases (GSK3β, CK1), the effect of XAV939 on cytosolic and nuclear presence of β-catenin in HUVEC was visualized using IF. Similar to overall β-catenin protein levels, a small but statistically significant decrease in nuclear β-catenin was observed (Fig. 1b). Within the nucleus, β-catenin associates with T cell factor/lymphoid enhancer factor (TCF/Lef) transcriptional complexes and thereby affects gene transcription, implying that XAV939 could attenuate β-catenin transcriptional activity in EC. However, nuclear β-catenin levels were generally found very low and the extent to which the relatively minor XAV939-induced decrease would affect β-catenin target gene transcription is unclear. In the case of canonical Wnt signaling, it has, however, been suggested that absolute levels of nuclear β-catenin are a poor indicator for target gene transcription. As such, to confirm whether the observed decrease affects β-catenin target gene transcription, a luciferase reporter gene assay for TCF/Lef activity (TOPFlash) was performed. Unfortunately, transfection methods to transiently transfect HUVEC with plasmid DNA were found to result in poor transfection efficiency or cell dedifferentiation, depending on the method used (lipofection or electroporation, respectively). As such, HEK cells were used for luciferase reporter gene assays. Here, XAV939 did not affect TFC/Lef-mediated transcription whereas β-catenin (CTNNB1) knockdown resulted in a marked decrease in TCF/Lef-mediated transcription. Conversely, treatment with lithium (LiCl), which inhibits GSK3β and thereby results stabilization of β-catenin, resulted in a marked increase (Fig. 1c). However, as β-catenin levels in HEK cells were generally found less affected by XAV939 compared to HUVEC (Fig. 1a), qPCR on well-known TCF/Lef target genes cyclin D1 (CCND1) and n-Myc (MYCN) was performed in HUVEC. Comparable to the TOPFlash results obtained in HEK cells, XAV939 did not decrease expression of either gene, whereas β-catenin knockdown did, indicating that the XAV939-induced decrease in β-catenin levels indeed does not affect TCF/Lef-mediated transcriptional activity. β-Catenin is, however, also a known nuclear interaction partner of forkhead box O3a (FOXO3a) which drives FOXO3a-mediated transcription (Tenbaum et al. 2012). As such, a FOXO3a luciferase reporter assay was performed (FHRE-Luc). Similar to the results from the TOPFlash assay, XAV939 did not affect FOXO3a transcriptional activity, while β-catenin knockdown or overexpression (β-catenin^S33Y^) did, further indicating that the XAV939-induced decrease in β-catenin likely does not contribute to transcriptional changes.

### XAV939 induces endothelial junctional remodeling and leakiness

**Fig. 2:**
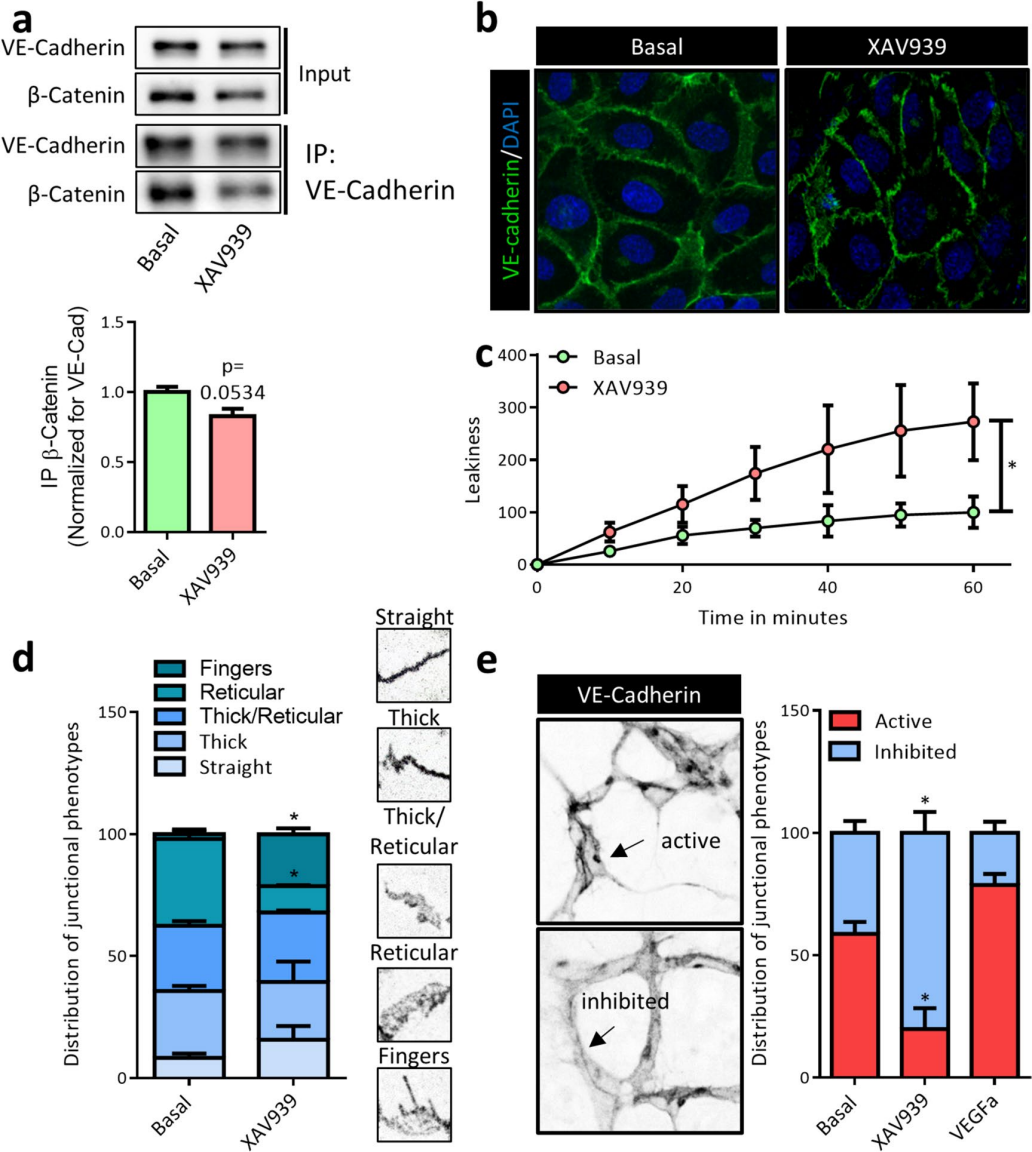
XAV939 treatment induces endothelial junctional remodeling and leakiness. **a** Representative WB images and quantification of β-catenin co-immunoprecipitation (co-IP) with VE-cadherin in HUVEC after treatment with XAV939 (5 µM, 6 h). For the input, 5% of the amount of protein used for co-IP was loaded. Data are normalized for the amount of VE-cadherin precipitated and to Basal and represent mean ± SEM of 3 independent experiments. **b** Representative confocal microscopy images of VE-cadherin (green) IF in HUVEC after XAV939 treatment (5 µM, 6h). Nuclei were counterstained with DAPI (blue). **c** Endothelial permeability assay of FITC-dextran. HUVEC were pre-incubated with XAV939 (5 µM, 6 h) after which FITC-dextran was added for 60 min to the top compartment. Fluorescence was measured in the bottom compartment at 10-min intervals. Data are normalized to the fluorescence signal after 60 min in the Basal condition and represent mean ± SEM of 5–6 independent measurements. **d** Analysis of cell junction morphology according to previous published work (Neto et al. 2018) and representative patches of the different junctional phenotypes in HUVEC treated with XAV939 (5 µM, 6 h) and labeled with VE-cadherin. Briefly, each image was divided into 16 patches and a morphological category was manually assigned to each patch (straight junctions, thick junctions, thick/reticular junctions, reticular junctions, and fingers). Data are expressed as a percentage of all patches analyzed and represent mean ± SEM of 3 pictures of 3 independent experiments. **e** Analysis of junctional phenotype in p5 explanted retina labeled with VE-cadherin and treated with XAV939 (5 µM, 16 h) and VEGF (50 ng/ml, 16 h) according to previously published work (Bentley et al. 2014) and representative images of typical examples of active and inhibited junctions. Bentley and colleagues originally defined 6 categories from highly inhibited to highly active. Here, we grouped categories 1–3 together as inhibited and categories 4–6 as active. An image of each retina was divided into 16 patches, and a morphological category was manually assigned to each patch. Data represent the distribution of patches and are expressed as mean ± SEM of 3 pictures of 3 explanted retina. *p < 0.05, n.s. *p* > 0.05 versus respective control condition

Since XAV939 had only a small effect on cytosolic β-catenin levels and no effect on TCF/Lef-mediated transcription, we next questioned whether it would affect the β-catenin pool at cell-cell junctions. Here, β-catenin associates with adherens junction (AJ) by coupling to VE-cadherin and α-catenin, forming a complex that is essential for stability of endothelial AJs. By immunoprecipitation (IP) of VE-cadherin, a small decrease in co-immunoprecipitated β-catenin was indeed observed after XAV939 treatment (Fig. 2a); however, this did not reach statistical significance. Quantification of the reverse immunoprecipitation of β-catenin was unreliable as a consequence of the slight reduction of β-catenin protein levels (Fig. 1a). Nonetheless, reduced β-catenin/VE-cadherin association could have consequences for normal functioning of cell-cell junctions in EC. Indeed, staining for VE-cadherin revealed a notable change in junctional phenotype after XAV939 treatment (Fig. 2b), where XAV939 results in a shift in VE-cadherin distribution from a continuous band with occasional reticular junctions to a distinctive discontinuous and serrated pattern. Such a pattern is associated with finger-like processes where the homophilic interaction between AJ proteins remain intact and gaps where these complexes detach (Li et al. 2016). Such a phenotypic shift would significantly impair the endothelial barrier function, allowing for intercellular transport between the apical and basal sides of an endothelial monolayer. Indeed, a Transwell in vitro experimental setup to study endothelial barrier function using FITC-dextran (70 kDa) revealed increased leakiness after XAV939 incubation (Fig. 2c). A more detailed analysis of the junctional phenotype has recently been defined (straight junctions, thick junctions, thick to reticular junctions, reticular junctions, and finger-like processes (Neto et al. 2018)). This classification revealed a redistribution of VE-cadherin resulting in a shift from reticular to serrated, finger-like junctions (Fig. 2d).

A prerequisite for angiogenesis and vascular remodeling to occur is that endothelial AJs undergo constant remodeling (Bentley et al. 2014), which consists of cycles of active remodeling and controlled destabilization, followed by maturation and inhibition of remodeling of junctional complexes. In this context, Bentley et al. have referred to serrated junctions as actively remodeling and straight junctions as inactive or inhibited. Here we employed a similar methodology in which explanted retina of p5 newborn mouse pups were cultured overnight. Confocal z-stacks of these retina were divided in a grid of square patches after which the VE-cadherin pattern in each patch was then classified as active or inhibited (Fig. 2e). Treatment with XAV939 resulted in a decrease in patches with predominantly active junctions whereas overnight treatment with the pro-angiogenic growth factor VEGF resulted in an increase in active patches, suggesting that XAV939 treatment inhibits junctional turnover during vascular remodeling. Together, these findings indicate that XAV939 leads to profound defects in endothelial AJ morphology and functioning.

### XAV939-induced junctional remodeling was accompanied by lowering total YAP1/TAZ levels

**Fig. 3:**
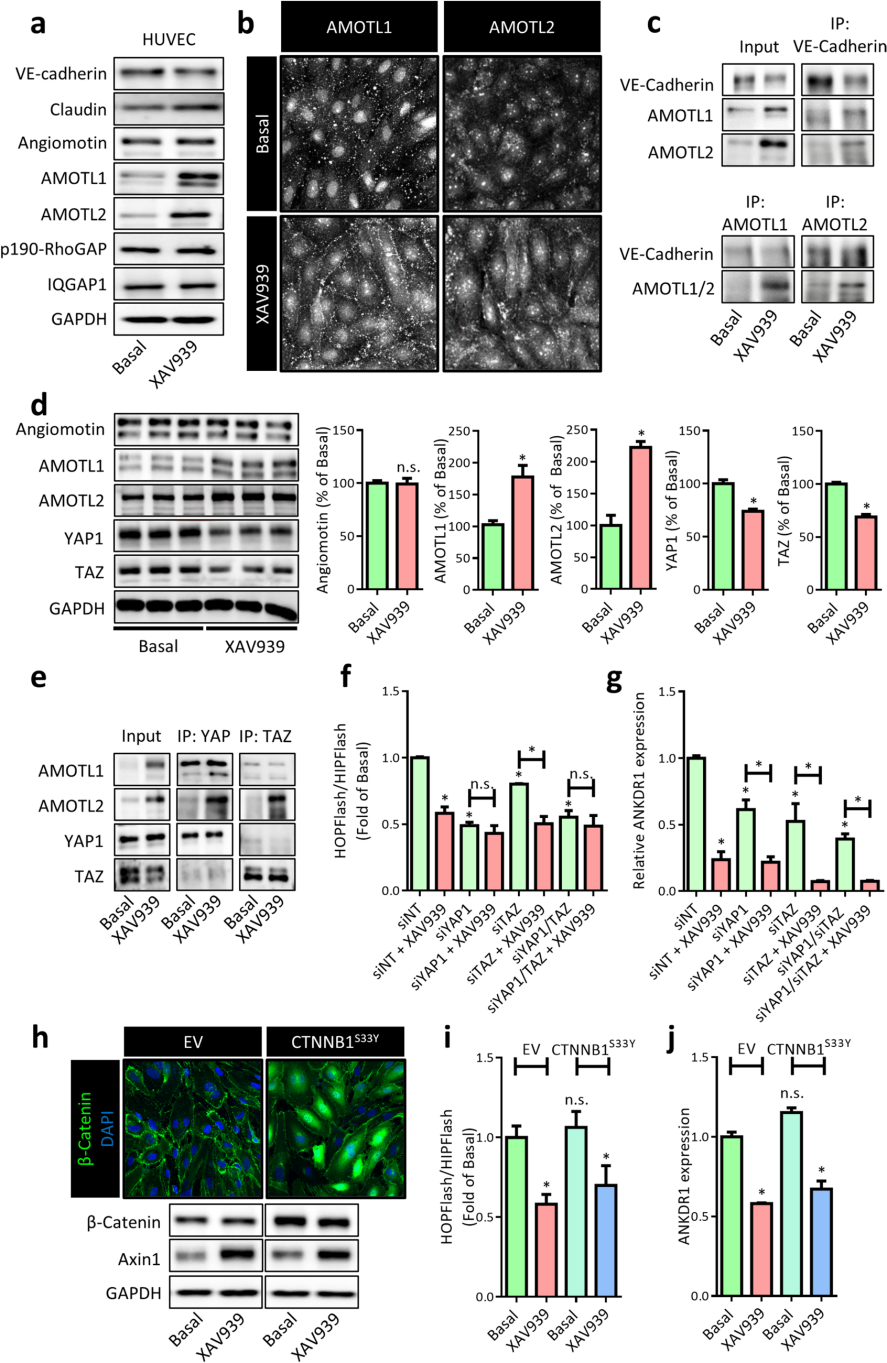
XAV939-induced junctional remodeling was accompanied by lowering total YAP1/TAZ levels. **a** Representative WB image of expression of different junctional proteins in HUVEC treated with XAV939 (5 µM, 6 h). **b** Representative confocal microscopy images of AMOTL1 and AMOTL2 IF in HUVEC after XAV939 treatment (5 µM, 6 h). **c** WB images of AMOTL1/2 co-IP with VE-cadherin and vice versa in HUVEC after treatment with XAV939 (5 µM, 6 h). For input 5% of the amount of protein used for co-IP was used. **d** Representative WB images and quantification angiomotin, AMOTL1, AMOTL2, YAP1, and TAZ protein levels in HUVEC after XAV939 treatment (5 µM, 6 h). Data are normalized to Basal and represent mean ± SEM of 3 independent experiments. **e** WB images of AMOTL1/2 co-IP with YAP1 and TAZ in HUVEC after treatment with XAV939 (5 µM, 6 h). For input 5% of the amount of protein used for co-IP was used. **f** TEAD luciferase reporter gene assay (HOPFlash) in HEK cells treated with XAV939 (5 µM, 24 h), YAP1 knockdown (siYAP1), TAZ knockdown (siTAZ), and the double YAP1/TAZ knockdown (siYAP1/siTAZ). Data are normalized for non-specific reporter gene activity (HIPFlash) and to the siNT control condition and represent mean ± SEM of 4 independent measurements. **g** qPCR for the TEAD target gene ANKDR1 expression in HUVEC treated with XAV939 (5 µM, 6 h), YAP1 knockdown (siYAP1), TAZ knockdown (siTAZ), and the double YAP1/TAZ knockdown (siYAP1/siTAZ). Data are normalized to non-targeting control siRNA (siNT) and represent mean ± SEM of 3 independent experiments. **h** Representative confocal microscopy images of β-catenin (green) IF in HUVEC expressing empty vector (EV) DNA (top left) and degradation-resistant β-catenin^S33Y^ (top right). Nuclei were counterstained with DAPI (blue). Representative WB images of β-catenin and axin1 protein expression in HUVEC after overexpression of β-catenin^S33Y^ and XAV939 treatment (5 µM, 6 h) are shown in the bottom. **i** TEAD luciferase reporter gene assay (HOPFlash) in HEK cells expressing empty vector (EV) DNA β-catenin^S33Y^ treated with XAV939 (5 µM, 24 h). Data are normalized for non-specific reporter gene activity (HIPFlash) and to the Basal control condition and represent mean ± SEM of 7 independent measurements. **j** qPCR for the TEAD target gene ANKDR1 expression in HUVEC expressing empty vector (EV) DNA and β-catenin^S33Y^ treated with XAV939 (5 µM, 6 h). Data are normalized to Basal and represent mean ± SEM of 3 independent experiments. *p < 0.05, n.s. *p* > 0.05 versus respective control condition or between conditions as indicated by brackets

As the observed XAV939-induced changes in endothelial AJs (Fig. 2) were not accompanied by a significant change in β-catenin protein levels or localization (Fig. 1), we next questioned whether XAV939 affects different components of the AJ complex that could underlie the phenotypic and functional changes. As such, we looked at expression levels of several known EC junctional proteins, namely, VE-cadherin, claudin, angiomotin, angiomotin-like 1 and 2 (AMOTL1 and AMOTL2, here referred to as AMOTL1/2), p190RhoGAP, and IQGAP1 (Bratt et al. 2005, Yamaoka-Tojo et al. 2006, Zheng et al. 2009, Zebda et al. 2013), and observed a strong increase in AMOTL1/2 protein levels (Fig. 3a). Angiomotin was originally identified as a regulator of EC migration and angiogenesis (Troyanovsky et al. 2001), but together with AMOTL1/2 has since been found to play important roles in proliferation, junctional maintenance, and cell polarity in many cell types. In vascular EC, loss of function of the different motins leads to impaired vascularization of tumors and to embryonic lethality during development (Holmgren et al. 2006, Aase et al. 2007, Levchenko et al. 2008). Further confirmation on localization of AMOTL1/2 in HUVECs confirmed that it is indeed localized at areas of cell-cell contact (Fig. 3b), where its involvement in tight-junction formation in EC is well established (Bratt et al. 2005, Zheng et al. 2009). However, although here we did not specifically distinguish between tight junctions and AJs for AMOTL1/2 localization, others have recently confirmed the involvement of motin family members in AJs, where they play a role in connecting radial actin filaments to the junctional complex (Hultin et al. 2014, Ortiz et al. 2015, Zheng et al. 2016, Hildebrand et al. 2017). Indeed, by co-IP we could confirm that AMOTL1/2 associates with VE-cadherin in HUVEC (Fig. 3c). Considering their function in mechanically coupling neighboring cells, several studies have addressed the involvement of AMOTL1/2 in mechanotransduction and have clearly characterized them as key regulators of YAP1/TAZ (Chan et al. 2011, Wang et al. 2011, Zhao et al. 2011). In line with these findings, we observed attenuated YAP1 and TAZ protein levels in response to XAV939 treatment in HUVEC (Fig. 3d). Interestingly, it appears that the XAV939-induced increase in AMOTL1 preferentially binds to YAP1 over TAZ, whereas AMOTL2 associates with both YAP1 and TAZ (Fig. 3e). The association between motins and YAP1 and TAZ has previously been shown to facilitate cytoplasmic and junctional retention (Chan et al. 2013), preventing nuclear translocation of YAP1 and TAZ. Within the nucleus, YAP1 and TAZ function as transcriptional co-activators by associating with TEAD-family transcription factors (Hooglugt et al. 2020). As such, we hypothesized that XAV939 suppresses TEAD-mediated gene transcription. Indeed, using the TEAD reporter gene assay (HOPFlash) in HEK cells, a strong reduction in TEAD reporter gene activity was observed (Fig. 3f). Similarly, YAP1 and TAZ knockdown (knockdown efficiency 60–70%, data not shown) decreased TEAD reporter gene activity. In line with this observation, a strong XAV939-induced decrease in expression of the well-known TEAD target gene ankyrin repeat domain-containing protein 1 (ANKDR1) was observed in HUVEC (Fig. 3g). Similar results were also obtained for the other well-known TEAD target genes connective tissue growth factor (CTGF, not shown), and cysteine-rich angiogenic inducer 61 (CYR61, not shown). As YAP1/TAZ and β-catenin signaling have been found to be heavily intertwined in which activation of Wnt/β-catenin promotes TEAD transcriptional activity (Rosenbluh et al. 2012, Azzolin et al. 2014, Benham-Pyle et al. 2015), we overexpressed a degradation-resistant mutant of β-catenin (β-catenin^S33Y^, Fig. 3h) to investigate whether the XAV939-induced regulation of YAP1/TAZ was dependent on β-catenin protein levels. This approach allowed us to confirm that the XAV939-induced decrease in TEAD reporter gene activity (Fig. 3i) as well as ANKDR1 expression in HUVEC (Fig. 3j) are independent of β-catenin protein levels.

### XAV939 increases VEGFR2 expression, but inhibits normal VEGFR2 trafficking

**Fig. 4:**
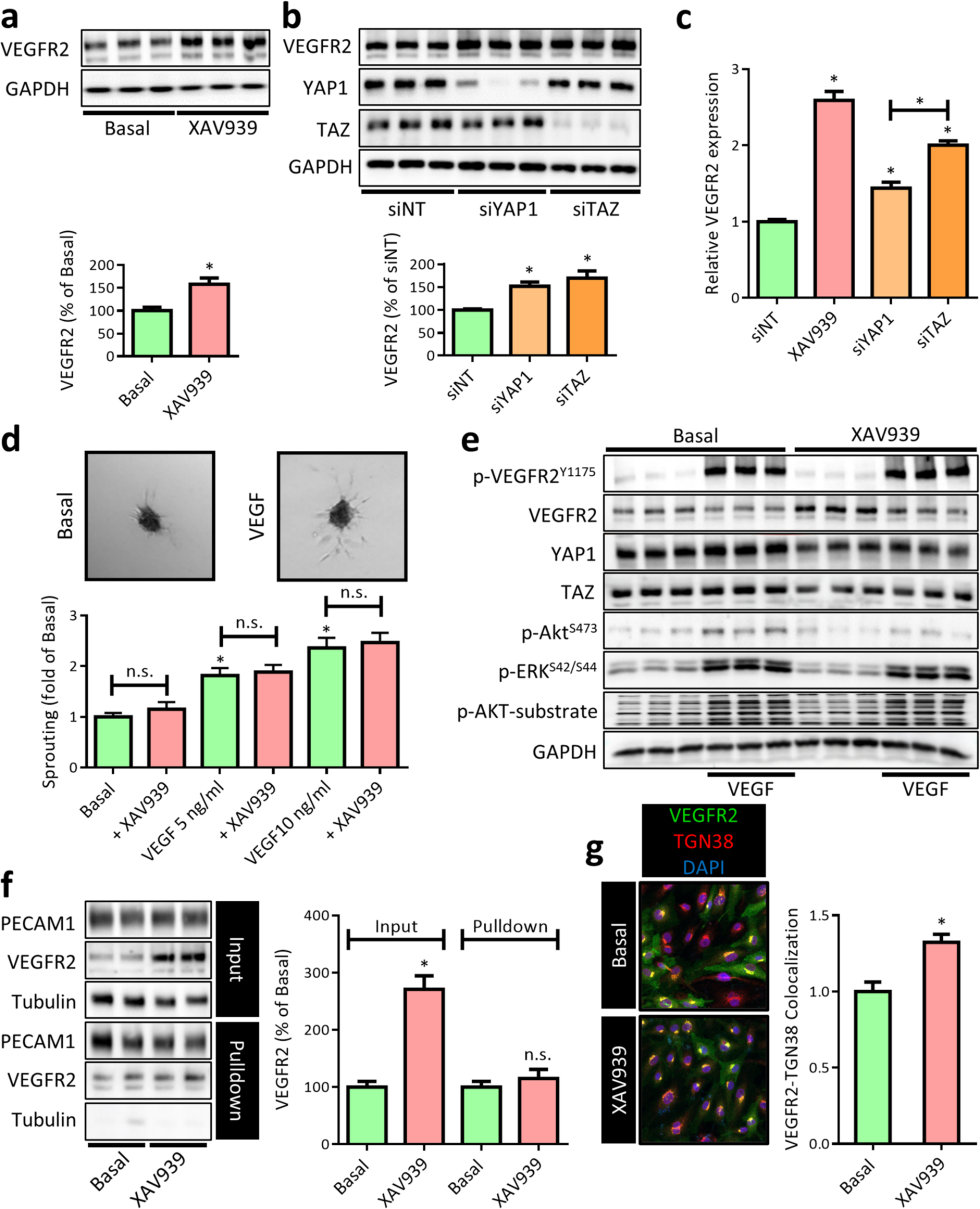
XAV939 treatment increases VEGFR2 expression, but inhibits normal VEGFR2 trafficking. **a** Representative WB images and quantification of VEGFR2 protein levels in HUVEC after XAV939 (5 µM, 6 h) treatment. Data are normalized to Basal and represent mean ± SEM of 3 independent experiments. **b** Representative WB images and quantification of VEGFR2 protein levels in HUVEC after YAP1 knockdown (siYAP1) and TAZ knockdown (siTAZ). Data are normalized to the siNT control condition and represent mean ± SEM of 3 independent measurements. **c** qPCR for VEGFR2 expression in HUVEC treated with XAV939 (5 µM, 6 h), YAP1 knockdown (siYAP1), and TAZ knockdown (siTAZ). Data are normalized to non-targeting control siRNA (siNT) and represent mean ± SEM of 3 independent experiments. **d** Quantification of endothelial spheroid sprouting assay in HUVEC. Spheroids were pre-incubated with XAV939 (5 µM, 16 h) after which spheroid were embedded in a collagen gel and stimulated with VEGF (50 ng/ml, 24 h). Representative pictures are shown above. The cumulative sprout length was measured. Data represent mean ± SEM of 16–18 spheroids from 3 independent experiments and are expressed normalized to the average cumulative sprout length from Basal. **e** WB images of VEGFR2, YAP1, and TAZ protein expression and phosphorylation status of VEGFR2 (Ser1175), AKT (Ser473), ERK1/2 (Ser42/44), and AKT substrate in HUVEC treated with XAV939 (5 µM, 6 h) and stimulated with VEGF (10 ng/ml, 15 min). **f** Representative WB images and quantification of platelet and endothelial cell adhesion molecule 1 (PECAM1) and VEGFR2 protein expression (input) and surface biotinylated PECAM1 and VEGFR2 in HUVEC after XAV939 treatment (5 µM, 6 h). PECAM1 was used as a surface control protein. Data are normalized to Basal and represent mean ± SEM of 3 independent experiments. **g** Representative confocal microscopy images of VEGFR2 (green) and the trans-Golgi marker TGN38 (red) and quantification of VEGFR2/TGN28 colocalization in HUVEC after XAV939 treatment (5 µM, 6 h). Nuclei were counterstained with DAPI (blue). Data are normalized to Basal and represent mean ± SEM of 6 independent experiments. *p < 0.05, n.s. *p* > 0.05 versus respective control condition or between conditions as indicated by brackets

During the vascularization of tissues, VEGF/vascular endothelial growth factor receptor 2 (VEGFR2) signaling is one of the major drivers of endothelial sprouting and cell-cell junction reorganization. Importantly, many of the transcriptional and functional effects of VEGF/VEGFR2 signaling involve activation of YAP1/TAZ (Wang et al. 2017, Elaimy and Mercurio 2018). As such, we questioned whether the observed changes in AJs (Fig. 2) involve defects in VEGF/VEGFR2 downstream YAP1/TAZ signaling. Surprisingly, protein expression of VEGFR2 increased in XAV939-treated HUVECs (Fig. 4a). Likewise, both YAP1 and TAZ knockdown increased VEGFR2 protein expression (Fig. 4b). As an accompanying increase in VEGFR2 mRNA was also detected, a transcriptional upregulation of VEGFR2 upon YAP1/TAZ depletion is likely (Fig. 4c). Nevertheless, as YAP1 and TAZ depletion is mostly associated with decreased VEGFR2 functionality (Elaimy and Mercurio 2018), we hypothesized that the increase in VEGFR2 expression is potentially a compensatory increase for this decreased functionality and thus does not enhance VEGF signaling. Indeed, using the well-established spheroid-based angiogenic sprouting assay (Heiss et al. 2015), we could confirm that the XAV939-induced increase in VEGFR2 expression does not enhance VEGF-induced sprouting angiogenesis (Fig. 4d). Upon binding of VEGF, VEGFR2 becomes phosphorylated at several intracellular tyrosine residues, of which VEGFR2^Y1175^ phosphorylation is indispensable for activation of downstream pathway. Indeed, no VEGF-induced increase in VEGFR2^Y1175^ phosphorylation after XAV939 treatment was observed (Fig. 4e), suggesting that there is no increased VEGR2 activation. In accordance, also no increase in the activation of the downstream kinases AKT (phospho-Ser473) or ERK1/2 (phosphor-Ser42 and Ser44) was detected. As these assays were performed under saturating VEGF concentrations without revealing an enhanced level of activated VEGFR2 (as assessed by residue Tyr1175 phosphorylation), the data suggest that the majority of the newly transcribed VEGFR2 were not exposed to VEGF. Of note, YAP1/TAZ have been described to be critical for retaining the VEGFR2 amount at the cell surface by regulating myosin 1C expression (Wang et al. 2017), which is crucial for trafficking of VEGFR from the Golgi apparatus (Tiwari et al. 2013). Indeed, using cell surface protein biotinylation, we could demonstrate that there was no increase in the amount of cell surface VEGFR2 despite the increase in total protein level (Fig. 4f). Consistently, co-staining of VEGFR2 and the Golgi marker TGN38 revealed an increase in VEGFR2 co-localization with TGN38 (Fig. 4g), confirming that the XAV939-induced increase in VEGFR2 accumulates in the Golgi apparatus and likely fails to reach the cell surface.

### XAV939 leads to defects in angiogenesis and endothelial migration

**Fig. 5:**
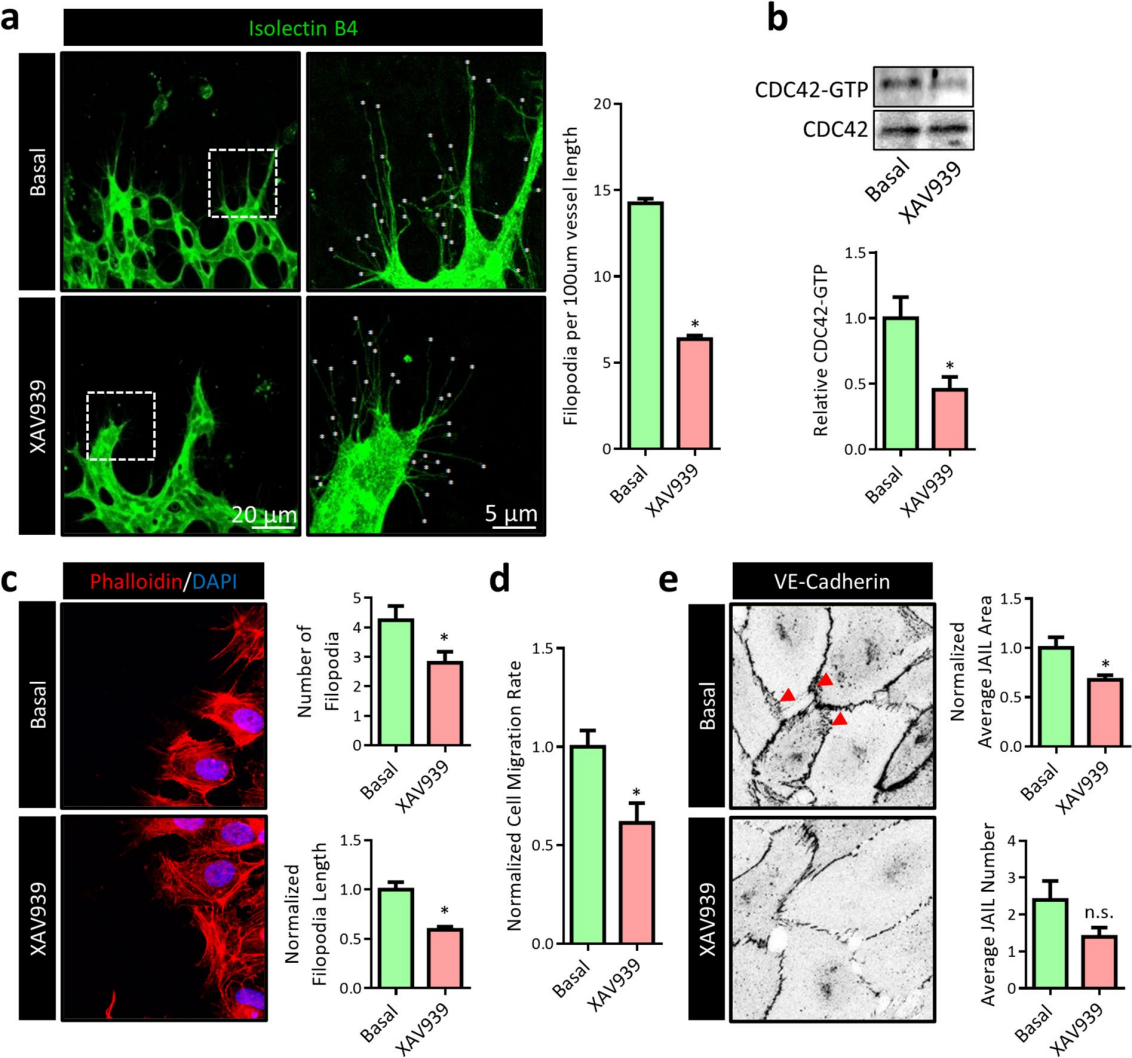
XAV939 treatment leads to defects in angiogenesis and endothelial migration. **a** Representative whole-mount confocal microscopy images of the vascular leading edge of explanted retinas from p5 newborn mice treated with XAV939 (5 µM, 16 h). Isolectin B4 (green) was used to stain the vasculature. The images on the right s are a magnification of the area indicated by the white boxes in the left images. Number of filopodia (indicated by white stars) were counted from 4 to 8 pictures per explanted retina. Data represent average number of filopodia per 100 µm vessel length and are expressed as mean ± SEM of 5 explanted retinas. **b** Representative WB images and quantification of a CDC42-GTP pulldown assay using GST-PAK1 beads in HUVEC treated with XAV939 (5 µM, 6 h). For input, 5% of the amount of protein used for CDC42-GTP pulldown was used. Data are normalized to CDC42 expression in the input and represent mean ± SEM of 4 independent experiments. **c** Representative confocal microscopy images of HUVEC at the migrating edge in a migration assay. Cells were allowed to migrate for 24 h in the presence or absence of XAV939 (5 µM) and aphidicolin (1 µg/ml). TRITC-phalloidin (red) was used to stain the actin cytoskeleton. Nuclei were counterstained with DAPI (blue). Number and length of filopodia per cell were quantified. Data represent average number of filopodia (top graph) and average length of filopodia per cell (bottom graph) and are expressed as mean ± SEM of 4–5 independent experiments. **d** Quantification of wound closure (migration rate) of HUVEC after 24 h of XAV939 treatment (5 µM) and aphidicolin (1 µg/ml). Data are normalized to Basal and represent mean ± SEM of 3 independent experiments. **e** Representative confocal microscopy images of VE-cadherin IF in HUVEC treated with XAV939 (5 µM, 6 h). Red arrowheads indicate JAIL. Average JAIL number per cell and area covered by JAIL were quantified. Data represent normalized JAIL area (top graph) and number of JAIL per cell and are expressed as mean ± SEM of 5 independent experiments. *p < 0.05, n.s. *p* > 0.05 versus respective control condition or between conditions as indicated by brackets

Angiogenesis is driven by a combination of proliferation, migration, and rearrangements of EC that require the turnover of AJs. Although in quiescent EC YAP1/TAZ signaling is inactivated, it is activated during vascular development and conditions that require angiogenesis. The critical involvement of YAP1/TAZ in the different processes of angiogenesis is well recognized (Kim et al. 2017), either in response to growth factor receptor signaling (as discussed above for VEGFR2) or by force-dependent remodeling of AJs (Giampietro et al. 2015) and is discussed in detail in a recent review by Hooglugt et al. (Hooglugt et al. 2020). Generally, deletion of endothelial YAP1/TAZ results in decreased number of angiogenic sprouts and accompanying tip cell numbers with defective filopodia (Kim et al. 2017). Using the ex vivo retina explant model (Sawamiphak et al. 2010), a marked decrease in the number of filopodia at the vascular front was observed after overnight treatment with XAV939 (Fig. 5a). As filopodia formation is regulated by the monomeric GTPase CDC42, an effector pulldown assay was performed to quantify the amount of active (GTP-bound) CDC42. As shown in Fig. 5b, XAV939 treatment strongly reduced GTP-bound CDC42. Although it is mechanistically unclear how XAV939 interferes with CDC42 activation, it is noteworthy that the involvement of TEAD-mediated transcription (Kim et al. 2017) as well as a cytoplasmic YAP1-regulated activation of CDC42 (Sakabe et al. 2017) have been described before in EC. Such a decrease in filopodia formation would functionally prevent directed cell migration. To study this, an in vitro cell migration assay was performed in combination with actin cytoskeleton visualization using fluorophore-conjugated phalloidin in HUVEC. Comparable to the ex vivo retinal explant data, XAV939 treatment strongly reduced the number and length of filopodia on the migrating edge (Fig. 5c), which was accompanied by decreased cell migration (Fig. 5d). Finally, the controlled turnover of endothelial AJs is an important requirement for cell migration during angiogenesis is. The formation and turnover of small actin-driven protrusions at areas of cell-cell contact have emerged as the driving force for controlled VE-cadherin dynamics and have been termed junction-associated intermittent lamellipodia (JAIL) (Cao et al. 2017). Interestingly, YAP1/TAZ have recently emerged as a key regulators of JAIL formation in EC. As shown in Fig. 5e, XAV939 treatment led to a marked reduction in both the number of JAIL as well as the area of cell-cell contact covered by JAIL, confirming that XAV939 indeed attenuates JAIL formation. Taken together, these findings indicate that the angiogenic defects induced by XAV939 phenotypically overlap with YAP1/TAZ-induced angiogenic defects, supporting the notion that XAV939 is a potent YAP1/TAZ inhibitor in EC.

### XAV939 induces radial actin filament formation

In EC, different distinct filamentous (F)-actin structures occur: the aforementioned structures present in filopodia and JAIL that rely predominantly on local activation of CDC42 and Rac1, but also junction-associated actin filaments such as cortical and radial actomyosin bundles that are predominantly regulated by RhoA (Cao and Schnittler 2019). Importantly, the anchorage of AJ proteins, including VE-cadherin, to the actin cytoskeleton plays a crucial role in the control of endothelial permeability and activation. During endothelial sprouting, AJs undergo changes in mechanical tension caused by formation of F-actin and actomyosin-mediated contractility (Phng et al. 2015). Interestingly, the different motin family members have been linked to formation of actin-cytoskeleton-based structures in epithelial cells (Ernkvist et al. 2006, Gagne et al. 2009). With regard to EC, AMOTL2 has been found to be required for proper connection of VE-cadherin to F-actin and passing down actomyosin-dependent forces at AJs (Hultin et al. 2014). Thus, we anticipated that XAV939 would alter the coupling of actomyosin filaments to VE-cadherin.

**Fig. 6:**
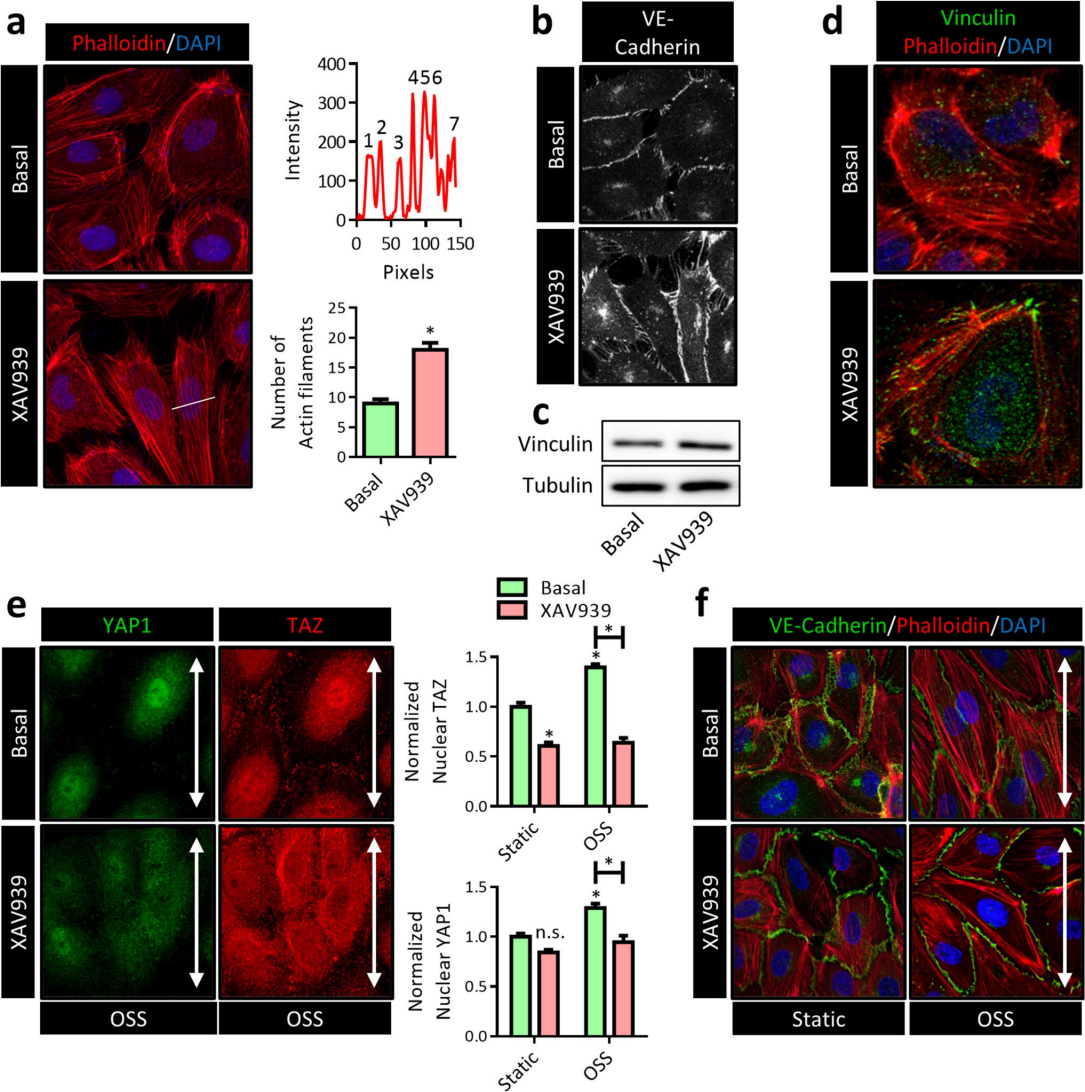
XAV939 treatment induces radial actin filament formation. **a** Representative confocal microscopy images of actin filaments in HUVEC treated with XAV939 (5 µM, 6 h). TRITC-phalloidin (red) was used to stain the actin cytoskeleton. Nuclei were counterstained with DAPI (blue). The number of actin filaments was quantified by counting the number of peaks from an intensity plot (top graph) derived from a cross section perpendicular to the direction of actin filaments (as indicated by the white line). Data (bottom graph) represent average number of actin filaments per cell and are expressed as mean ± SEM of 5 independent experiments. **b** Representative confocal microscopy images of VE-cadherin IF in HUVEC treated with XAV939 (5 µM, 6 h). **c** Representative WB image of vinculin protein expression in HUVEC after XAV939 treatment (5 µM, 6 h). **d** Representative confocal microscopy images of actin filaments (red) and vinculin (green) in HUVEC treated with XAV939 (5 µM, 6 h). Nuclei were counterstained with DAPI (blue). **e** Representative confocal microscopy images of YAP1 (green) and TAZ (red) IF in HUVEC treated with XAV939 (5 µM, 6 h) cultured under oscillatory shear stress (OSS, 12 dyne/cm^2^ at 2 Hz, direction of flow indicated by white arrowheads). Nuclei were counterstained with DAPI (channel not shown in images) for quantification of nuclear YAP1/TAZ. Data in graphs represent nuclear TAZ (top graph) and YAP1 (bottom graph) and are expressed normalized to static Basal and represent mean ± SEM of 4–7 independent experiments. **f** Representative confocal microscopy images of VE-cadherin (green) and actin filaments (red) IF in HUVEC treated with XAV939 (5 µM, 6 h) cultured under oscillatory shear stress (OSS, 12 dyne/cm^2^ at 2 Hz, direction of flow indicated by white arrowheads). Nuclei were counterstained with DAPI (blue). *p < 0.05, n.s. *p* > 0.05 versus respective control condition or between conditions as indicated by brackets

Generally, in quiescent endothelium, linear VE-cadherin junctions are in close proximity to thick cortical actin bundles that are believed to help to stabilize AJs. In contrast, during vascular remodeling perpendicular-oriented radial actomyosin bundles that terminate at VE-cadherin sites are observed (Dorland and Huveneers 2017). Indeed, XAV939 treatment induced a marked increase in the number of radial F-actin bundles as well as a loss of cortical actin (Fig. 6a). Interestingly, in EC actomyosin-generated force exerted on AJs with concomitant loss of cortical actin results in an interrupted VE-cadherin pattern which is reminiscent of VE-cadherin fingers (Fig. 6b) (Hayer et al. 2016). This junctional phenotype, which is defined by radial F-actin bundles at sites of junctional remodeling, has more recently been termed focal adhesion junctions (FAJs) as vinculin is recruited to these structures by the force-dependent unfolding of α-catenin, exposing a vinculin binding site (Huveneers et al. 2012, Kotini et al. 2022). Expression of vinculin was slightly increased in EC treated with XAV939, although this did not reach statistical significance (Fig. 6c). More strikingly, and reminiscent to FAJs, vinculin foci were detected at sites where radial F-actin bundles were perpendicular to cell-cell contacts after XAV939 treatment, confirming that XAV939 promoted vinculin recruitment to sites of AJ remodeling (Fig. 6d). Interestingly, under flow-related shear stress, the endothelial AJ acts as a mechanosensor that links mechanical strain to cellular adaptations, in which YAP1/TAZ are among the most prominent transducers of shear stress in EC. In fact, the recent observation that endothelial shear stress induces nuclear import of YAP1 independent of Hippo signaling is of particular interest here (Nakajima et al. 2017). This work elegantly describes how YAP1 is kept in the cytoplasm by binding to angiomotin in static EC, but is released from angiomotin that preferentially binds to F-actin upon shear stress. In accordance, nuclear YAP1 and TAZ localization was markedly increased in HUVEC cultured under oscillatory shear stress (OSS, 12 dyne/cm^2^ at 2 Hz, direction of flow indicated by white arrowhead) (Fig. 6e). This increase in nuclear YAP1 and TAZ was fully prevented by XAV939, suggesting that the XAV939-induced increase in AMOTL1/2 (and subsequent decrease in YAP1 and TAZ) can overcome the described F-actin-induced release of YAP1 from angiomotin. Furthermore, shear stress induces an alignment of cells in endothelial monolayers in the direction of the flow; however, YAP1- and TAZ-depleted cells are deficient in this response (Wang et al. 2016b). In line with these observations, cells subjected to a combination of XAV939 and OSS fail to align in the direction of the flow (Fig. 6f, direction of flow indicated by white arrowheads), further supporting the notion that XAV939 treated cells are deficient in functional YAP1/TAZ signaling.

### The endothelial actin cytoskeleton differentially regulates YAP1 and TAZ

Remodeling and stability of AJs are tightly controlled by monomeric GTPases of the Rho family that modulate cytoskeletal dynamics, in which the activation of RhoA promotes Rho-dependent kinase (ROCK)-induced actomyosin-mediated junctional tension (van Buul and Timmerman 2016). It is well-established that mechanical cues that converge on the actin cytoskeleton, such as junctional tension, determine YAP1/TAZ activity (Seo and Kim 2018). Studies on predominantly cells of epithelial origin have convincingly demonstrated that induction of F-actin bundling promotes nuclear import of YAP1/TAZ, while in contrast, interfering with F-actin bundling causes retention of YAP1/TAZ in the cytoplasm (Dupont et al. 2011). It is assumed that high actomyosin tension results in increased nuclear import of YAP1 by Hippo-dependent and independent mechanisms (Seo and Kim 2018). On a first glance, these observations are apparently in contrast with our findings that suggest XAV939-induced YAP1/TAZ inhibition is accompanied by F-actin bundling (Fig. 6).

**Fig. 7:**
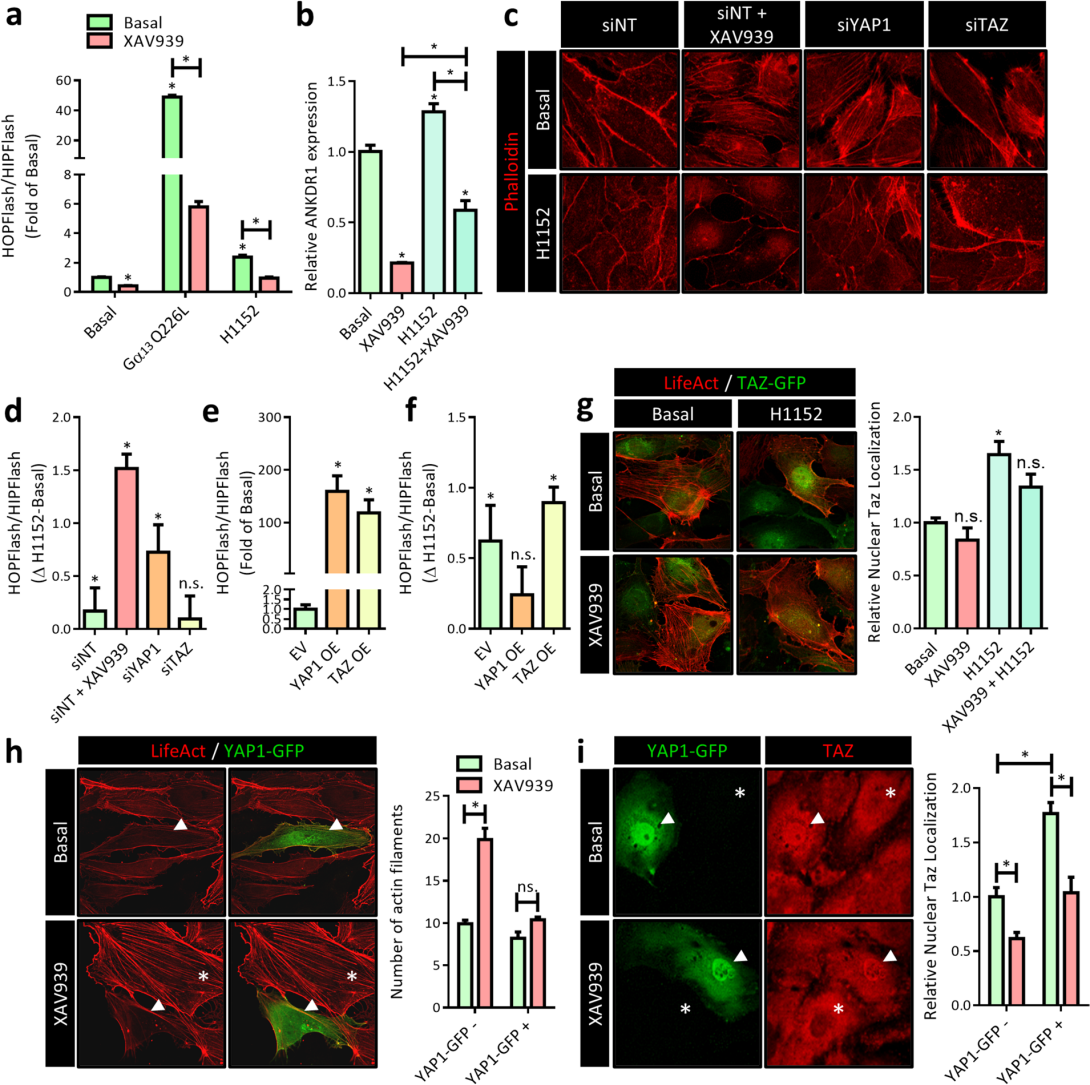
The endothelial actin cytoskeleton differentially regulates YAP1 and TAZ. **a** TEAD luciferase reporter gene assay (HOPFlash) in HEK cells treated with XAV939 (5 µM, 24 h). The constitutively active Gα13 mutant Gα13Q226L was expressed to activate RhoA/ROCK. The H1152 (1 µM, 24 h) compound was used to block ROCK activity. Data are normalized for non-specific reporter gene activity (HIPFlash) and to the Basal empty vector (EV) control condition and represent mean ± SEM of 4 independent measurements. **b** qPCR for the TEAD target gene ANKDR1 expression in HUVEC treated with XAV939 (5 µM, 6 h) and the ROCK inhibitor H1152 (1 µM, 6 h). Data are normalized to Basal and represent mean ± SEM of 3 independent experiments. **c** Representative confocal microscopy images of actin filaments after H1152 (1 µM, 6 h) treatment in HUVEC treated with XAV939 (5 µM, 6 h) or after YAP1 knockdown (siYAP1) and TAZ knockdown (siTAZ). TRITC-phalloidin (red) was used to stain the actin cytoskeleton. **d** TEAD luciferase reporter gene assay (HOPFlash) after H1152 (1 µM, 24 h) treatment in HEK cells treated with XAV939 (5 µM, 24 h) or after YAP1 knockdown (siYAP1) and TAZ knockdown (siTAZ). Data are normalized for non-specific reporter gene activity (HIPFlash) and to the siNT control condition and are expressed as the delta between H1152-treated and untreated cells and represent the mean ± SEM of 4 independent measurements. **e** TEAD luciferase reporter gene assay (HOPFlash) in HEK cells overexpressing YAP1-GFP (YAP1 OE) or TAZ-GFP (TAZ OE). Data are normalized for non-specific reporter gene activity (HIPFlash) and to the empty vector (EV) control condition and represent the mean ± SEM of 4 independent measurements. **f** TEAD luciferase reporter gene assay (HOPFlash) after H1152 (1 µM, 24 h) treatment in HEK cells overexpressing YAP1-GFP (YAP1 OE) or TAZ-GFP (TAZ OE). Data are normalized for non-specific reporter gene activity (HIPFlash) and to the empty vector (EV) control condition and are expressed as the delta between H1152-treated and untreated cells and represent the mean ± SEM of 4 independent measurements. **g** Representative confocal microscopy images of TAZ (green) in HUVEC overexpressing TAZ-GFP treated with XAV939 (5 µM, 6 h) and H1152 (1 µM, 6 h). The actin cytoskeleton was visualized by co-expressing LifeAct-mRuby2. Nuclei were counterstained with DAPI (channel not shown in images) for quantification of nuclear TAZ. Data in graphs represent nuclear TAZ and are expressed normalized to Basal and represent mean ± SEM of 5 independent experiments. **h** Representative confocal microscopy images of YAP1 (green) in HUVEC overexpressing YAP1-GFP treated with XAV939 (5 µM, 6 h). The actin cytoskeleton was visualized by co-expressing LifeAct-mRuby2. In the left images the green YAP1 channel is removed for improved visibility of the actin cytoskeleton. The white arrowheads indicate cells overexpressing YAP1-GFP. The white asterisks indicate cells without YAP1-GFP overexpression. Number of actin filaments were quantified by counting the number actin filaments. Data represent average number of actin filaments per cell and are expressed as mean ± SEM of 3 independent experiments. **i** Representative confocal microscopy images of YAP1 (green, left images) and TAZ (red, right images) IF in HUVEC overexpressing YAP1-GFP treated with XAV939 (5 µM, 6 h). In the left images, the red TAZ channel is removed; in the right images, the green YAP1 channel is removed. Nuclei were counterstained with DAPI (channel not shown in images) for quantification of nuclear TAZ. The white arrowheads indicate cells overexpressing YAP1-GFP (YAP1-GFP +). The white asterisks indicate cells without YAP1-GFP overexpression (YAP1-GFP −). Data in graphs represent nuclear TAZ and are expressed normalized to Basal in YAP1-GFP − cells and represent mean ± SEM of 3 independent experiments. *p < 0.05, n.s. *p* > 0.05 versus respective control condition or between conditions as indicated by brackets

To investigate the interaction between the cytoskeleton and YAP1/TAZ further, we increased RhoA/ROCK activity by expressing a constitutively active mutant of the G protein Gα_13_ (Q226L), which is known to induce a robust F-actin assembly. Indeed, expression of Gα_13_Q226L resulted in the expected increase in TEAD-mediated transcription in HEK cells, whereas XAV939 treatment prevented this Gα_13_Q226L-induced increase (Fig. 7a). Although both Gα_13_Q226L and XAV939 promote F-actin bundling and mechanical tension, we assume that the XAV939-induced decrease in YAP1/TAZ protein levels, as a consequence of increased AMOTL1/2 stability, prevents the increase in TEAD-mediated transcription that would be expected with increased F-actin bundling. However, surprisingly, we found that the selective ROCK inhibitor H1152, which prevents F-actin bundling and mechanical tension (Breyer et al. 2012), also results in an increase in TEAD-mediated gene transcription (Fig. 7a). These data indicate that both the increase and decrease in F-actin bundling and mechanical tension have the potential to promote YAP1/TAZ transcriptional activity. In line with this interpretation, the XAV939-induced downregulation of ANKDR1 was partially prevented by H1152 in HUVEC (Fig. 7b), confirming that preventing F-actin bundling might in fact support the transcriptional activity of YAP1/TAZ in EC. It is important to note that ECs are always cultured in high-density, tightly packed monolayers and recent observations in keratinocytes have demonstrated that in high-density cells, actomyosin-based tension at AJs causes cytoplasmic retention of YAP1/TAZ, whereas in lower-density cells, this results in nuclear import (Hirata et al. 2017).

However, from our so far obtained results, it is still unclear whether the decrease in H1152-induced TEAD-mediated transcription by XAV939 (Fig. 7a, b) is the consequence of a decrease in YAP1/TAZ or XAV939-induced F-actin bundling. Thus, we performed phalloidin staining to visualize the actin cytoskeleton and observed that the XAV939-induced radial F-actin bundling was indeed prevented by H1152 (Fig. 7c). These data indicate that XAV939 interferes with H1152-induced TEAD-mediated transcription on the level of YAP1/TAZ and not the actin cytoskeleton. Interestingly, depleting YAP1 or TAZ independently using siRNA revealed that YAP1 depletion also enhances radial F-actin bundling and lowers cortical actin (Fig. 7c), comparable to XAV939 (Fig. 6), suggesting that the interaction between YAP1 and the actin cytoskeleton is bidirectional. This observation is in line with earlier observation that demonstrated that endothelial YAP1/TAZ depletion promotes F-actin bundling (Neto et al. 2018). Although the underlying mechanism has not been elucidated in EC, in epithelial cells reverse regulation of the actin cytoskeleton by YAP1 depletion has been reported. It was found that nuclear YAP1 induces the expression of the Rho GTPase activating proteins (RhoGAP) ARHGAP18/29, thereby deactivating RhoA/ROCK (Qiao et al. 2017). Although the siRNA-mediated knockdown of TAZ in EC was very effective in our hands (see Fig. 4b), surprisingly, its depletion did not prevent radial F-actin bundling (Fig. 7c), suggesting that YAP1 and TAZ are functionally not completely overlapping. We thus further studied the effect of H1152 on TEAD-mediated transcription in YAP1- and TAZ-depleted cells and observed that H1152 rescued TEAD-mediated transcription in XAV939-treated and YAP1-depleted cells. However, in TAZ depleted cells, TEAD-mediated transcription was not different in the absence or presence of H1152 (Fig. 7d), which suggests that YAP1 and TAZ differentially interact with the cytoskeleton. Next, we were interested if the reverse would occur in YAP1- or TAZ-overexpressing cells; thus, H1152 would promote TEAD-mediated transcription in TAZ-overexpressing but not in YAP1-overexpressing cells. Indeed, while both YAP1 and TAZ overexpression caused the expected increase in TEAD-mediated transcription (Fig. 7e), H1152 further enhanced TEAD-mediated transcription in only TAZ-overexpressing cells (Fig. 7f). Therefore, we hypothesized that a release of mechanical tension by preventing radial F-actin bundling could promote nuclear import of TAZ. Indeed, when we investigated TAZ localization in HUVECs, we observed a marked increase in nuclear TAZ after H1152 treatment (Fig. 7g). Furthermore, as both XAV939 and YAP1 depletion cause increased radial F-actin bundling, this would further imply that YAP1 overexpression could reverse XAV939-induced radial F-actin bundling. Indeed, we observed that HUVEC-overexpressing YAP1 (Fig. 7h, white arrowheads, YAP1-GFP+ cells) are protected from the XAV939 effect on the actin cytoskeleton (Fig. 7h, white asterisks, YAP1-GFP- cells). As such, we additionally hypothesized that TAZ nuclear localization would be induced in YAP1-overexpressing cells. Indeed, we observed increased TAZ nuclear localization in YAP1-overexpressing EC (Fig. 7i, white arrowheads, YAP1-GFP+ cells). Furthermore, YAP1 overexpression fully prevented the XAV939-induced decrease in TAZ nuclear localization. Taken together, these findings suggest that YAP1 and TAZ interact differentially with the actin cytoskeleton, in which YAP1 functions both upstream and downstream of the actin cytoskeleton, whereas TAZ appears to function only downstream.

### Proposed mechanistical model of XAV939-induced YAP1/TAZ inhibition

Thus far, our findings indicated altered F-actin bundling, YAP1/TAZ upregulation, and differential localization as underlying causes for the EC defects occurring upon tankyrase inhibition. Taking into account also data reported in the literature on the interaction of YAP1/TAZ with the actin cytoskeleton, we propose the following model by which tankyrase inhibitors affect EC function through regulation of YAP1/TAZ.

**Fig. 8:**
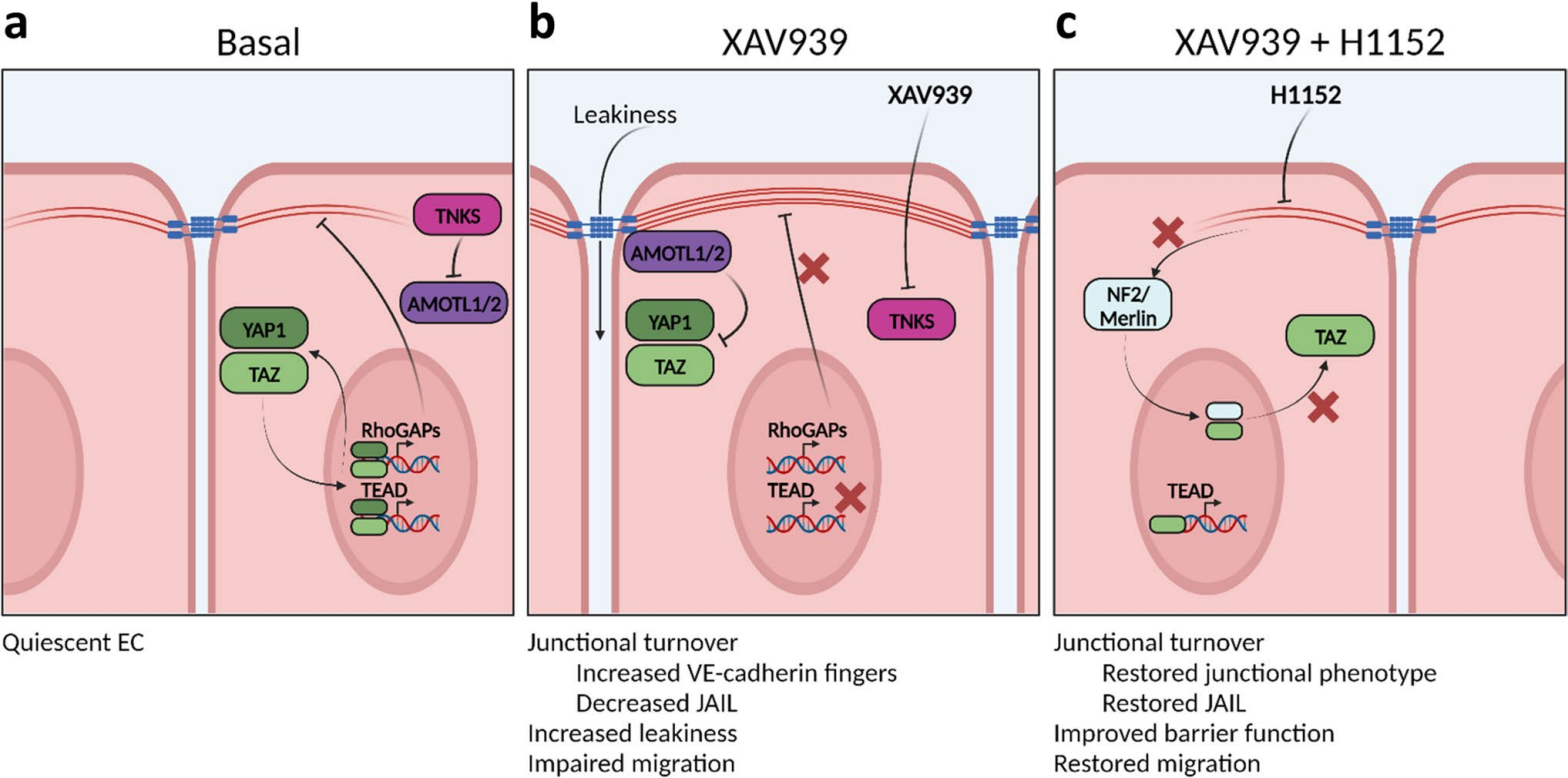
Proposed hypothetical model on the mechanism of action of XAV939-induced YAP1/TAZ inhibition. **a** Under basal conditions, AMOTl1/2 levels are suppressed by tankyrases (TNKS). Cytosolic and nuclear YAP1 and TAZ are in equilibrium with the majority being localized within the cytosol. Nuclear YAP1 and TAZ regulate expression of TEAD target genes, but also of several RhoGAPs (e.g., ARHGAP29, DLC1) that regulate actin dynamics. **b** In the presence of XAV939, TNKS are inhibited, resulting in increased stability and junctional recruitment of AMOTL1/2, which suppresses YAP1/TAZ nuclear translocation and TEAD-mediated transcription. In turn, this leads to increased junctional tension by F-actin bundling, presumably by a decrease in expression and/or activity of RhoGAPs. As a consequence, AJs open allowing for increased paracellular leakiness. Further, the increase in junctional tension impairs actin dynamics required for migration of cells. **c** Inhibition of XAV939-induced junctional F-actin bundling using the ROCK inhibitor H1152 restores endothelial barrier function and migratory capacity. Further, H1152 also leads to increased nuclear localization of TAZ. Based on studies in epithelial cells, we propose that for EC increased junctional F-actin tension releases NF2/merlin, which subsequently drives nuclear export of TAZ. Thus, by decreasing junctional F-actin tension, H1152 enhances nuclear localization of TAZ in EC. This figure was created with BioRender.com

Under basal conditions (Fig. 8a), ECs are quiescent and have stable cell-cell junctions and a well-functioning endothelial barrier function. The basal tankyrase activity prevents AMOTL1/2 accumulation by promoting AMOTL1/2 ubiquitination by the E3 ligase RNF146 (Wang et al. 2015). Cytosolic and nuclear YAP1 and TAZ exist in equilibrium where the majority is present within the cytosol. This equilibrium reinforces quiescence and controlled junctional turnover and maintenance by promoting a basal level of TEAD-mediated transcription and expression of RhoGAPs (such as ARHGAP29 in epithelial cells (Qiao et al. 2017) and DLC1 in EC (van der Stoel et al. 2020)) that prevent actomyosin tension on junctional complexes.

In the presence of the tankyrase inhibitor XAV939 (Fig. 8b), AMOTL1/2 protein stability is increased and consequently, as AMOTL1/2 act as scaffolds to promote the YAP1 and TAZ localization to the cytoplasm, cell junctions, and actin cytoskeleton (Chan et al. 2011, Dai et al. 2013), this results in a robust inhibition of YAP1/TAZ nuclear translocation and TEAD-mediated transcription (Dasgupta and McCollum 2019). Further, expression of RhoGAPs is decreased and as such, actomyosin-mediated tension is increased. Indeed, our findings reported herein, support increased F-actin bundling as a result of XAV939-mediated inhibition of YAP1/TAZ. Consequently, XAV939 disrupts AJs and lowers the barrier function, resulting in increased leakiness. This is further consistent with the current understanding of barrier function regulation in EC, where VE-cadherin clustering induces Rac1-mediated recruitment of RhoGAPs such as ARHGAP29 to AJs (Wildenberg et al. 2006, Post et al. 2013). Vice versa, an increase in actomyosin contractility has been shown to restrict JAIL formation in EC (Breslin et al. 2016), which is consistent with our observations.

It is generally believed that high F-actin levels sequester AMOTL1/2 by binding to F-actin, and thus, they are unable to regulate YAP1/TAZ (Mana-Capelli et al. 2014). Indeed, shear stress has been shown to induce AMOT sequestration to F-actin, which allows YAP1 to enter the nucleus (Nakajima et al. 2017). As we observe increased nuclear TAZ after F-actin disruption by the ROCK inhibitor H1152, our data are apparently in disagreement with this well-established regulation of YAP1/TAZ by the actin cytoskeleton. However, it is important to consider that studies showing increased YAP1/TAZ activity after F-actin bundling observe this in response to mechanical tension originating from extracellular matrix-cell adhesion sites, mediated via focal adhesions in low-density cell culture, and not mechanical tension between adjacent cells in a tight EC monolayer, which is mediated via AJs. The underlying mechanisms that could explain this discrepancy are not fully elucidated, but in high-density epithelial cells a similar response was observed (Furukawa et al. 2017). F-Actin organization shifts from stress fibers to circumferential F-actin belts between low and high densities. Interestingly, this work demonstrated that in high cell density, the protein NF2/merlin is released from AJs where it localizes in low cell density (Gladden et al. 2010) by increased junctional tension. The released NF2/merlin subsequently promotes nuclear export of YAP1/TAZ via its nuclear export signal (Furukawa et al. 2017). We therefore hypothesize that the H1152-induced increase in nuclear TAZ and TEAD-mediated transcription is the consequence of a release of junctional tension and thereby sequestration of NF2/Merlin at AJs (Fig. 8c). Interestingly, NF2/merlin interacts with and is controlled by motin family members such as AMOTL1/2 (Li et al. 2015), it is therefore likely that the XAV939-induced increase in AMOTL1/2 regulates YAP1/TAZ on multiple levels in EC.

### Inhibition of F-actin bundling counteracts XAV939-induced alterations in EC

**Fig. 9:**
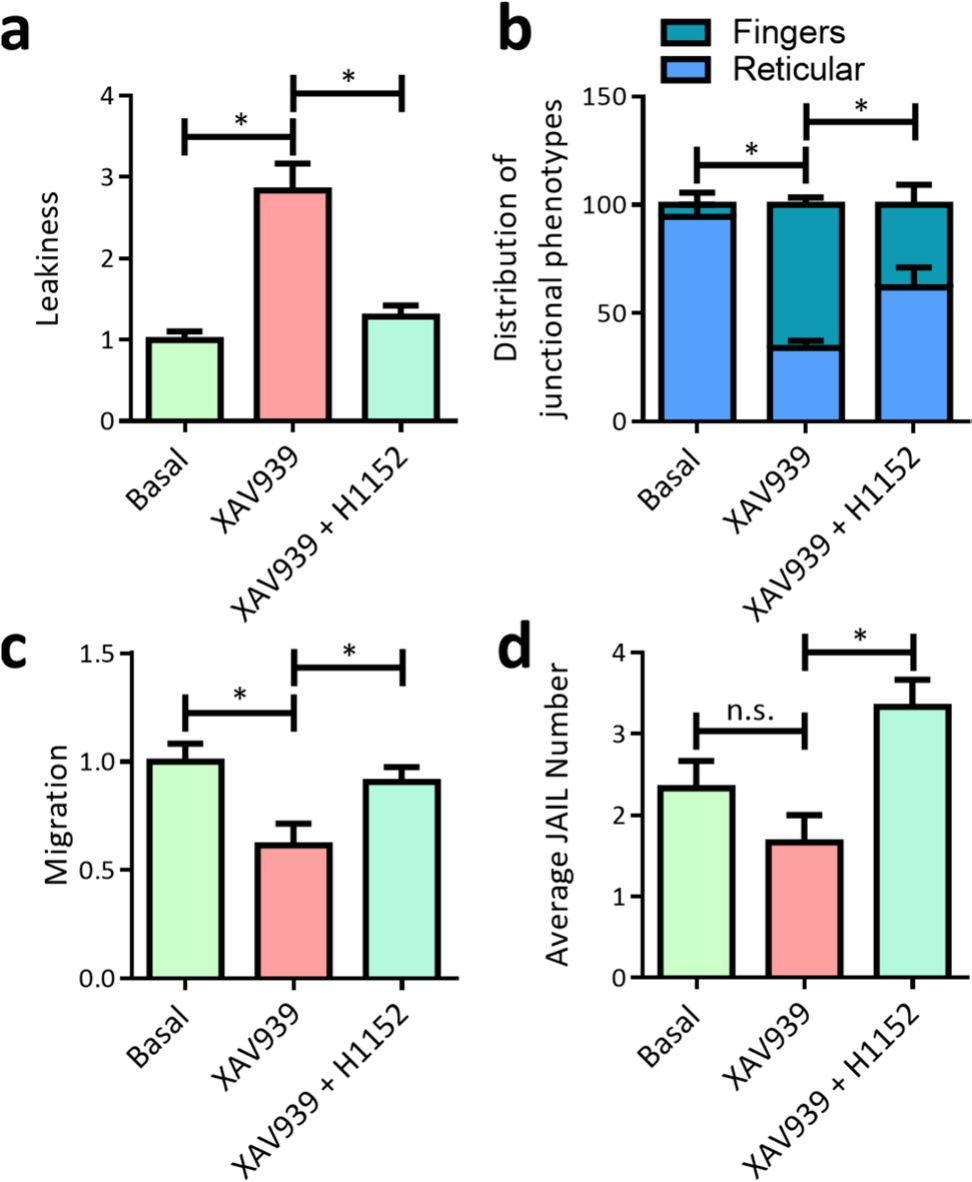
Inhibition of F-actin bundling counteracts XAV939-induced alterations in EC. **a** Endothelial permeability assay as in Fig. 2a. HUVEC were pre-incubated with XAV939 (5 µM, 6 h) and H1152 (1 µM, 6 h) after which FITC-dextran was added to the top compartment. Data represent the fluorescent signal in the bottom compartment after 1 h and are normalized to the Basal condition and represent mean ± SEM of 5 independent measurements. **b** Analysis of cell junction morphology as in Fig. 2d. HUVEC were treated with XAV939 (5 µM, 6 h) and H1152 (1 µM, 6 h). Data are expressed as a percentage of all patches analyzed and represent mean ± SEM of 3 pictures of 3 independent experiments. **c** Quantification of wound closure (migration rate) as in Fig. 5d in HUVEC treated with XAV939 (5 µM, 24 h) and H1152 (1 µM, 24 h). Data are normalized to Basal and represent mean ± SEM of 3 independent experiments. **d** Analysis of JAIL number per cell as in Fig. 5e in HUVEC treated with XAV939 (5 µM, 24 h) and H1152 (1 µM, 24 h). Data represent average number of JAIL per cell and are expressed as mean ± SEM of 3 independent experiments. *p < 0.05, n.s. *p* > 0.05 versus respective control condition or between conditions as indicated by brackets

According to our model, preventing F-actin bundling should counteract the XAV939-induced functional alterations in EC described above, like the increase in endothelial monolayer leakiness (Fig. 2c), the switch in the junctional phenotype from reticular to finger-like junctions (Fig. 2d), the attenuated migration rate (Fig. 5d), and the reduction in JAIL number per cell (Fig. 5e). We therefore studied whether a combined treatment of HUVEC with H1152 and XAV939 would counteract the tankyrase inhibition-induced defects. Indeed, H1152 fully prevented XAV939-induced endothelial monolayer leakiness (Fig. 9a), partially restored the junctional phenotype (Fig. 9b), and fully restored the endothelial migration capacity (Fig. 9c) as well as JAIL formation (Fig 9d). Apparently, all studied XAV939-induced endothelial functional defects are dependent on F-actin bundling. However, from these observations we cannot conclude whether the restoration of EC function by H1152 requires a protective role of TAZ nuclear translocation or whether counteracting XAV939-induced F-actin bundling alone and the resulting increase in mechanical tension by itself is already sufficient.

## Discussion

### Tankyrase inhibitions affects YAP1/TAZ signaling and the actin cytoskeleton in the endothelium

Tankyrase inhibitors have recently emerged as a new class of anti-tumorigenic drugs by their virtue of potently inhibiting Wnt/β-catenin signaling in malignant cells with high intrinsic β-catenin activity that are considered for clinical use. A first-in-human study has been recently reported for stenoparib (E7449 (Plummer et al. 2020)) which is currently in a phase 2 trial for treatment of ovarian cancer. However, the effects of tankyrase inhibition are not limited to malignant cells but also affect tissues and organs throughout the body. Especially its effect on the EC lining the vasculature requires consideration, which to date has only been addressed in a few studies (Jean LeBlanc et al. 2019, Zeng et al. 2021, Zhong et al. 2021). Additionally, it is important to note that tankyrases have recently been implicated as regulators of other major signaling pathways, most notably the YAP1/TAZ (Wang et al. 2015, Wang et al. 2016a), and the abovementioned studies did not further extend on the underlying signaling involved but assumed any effects were the consequence of impaired β-catenin signaling. Therefore, we aimed to study the effect of tankyrase inhibition on EC function and the underlying signal transduction involved using the prototypical tankyrase inhibitor XAV939. We observed significant XAV939-induced alterations in endothelial AJ morphology with an increase in finger-like junctions, leading to increased leakiness. Although β-catenin levels were slightly lower in cells treated with XAV939, this had no consequence for TCF/Lef-mediated transcription. Since tankyrase inhibition has already been implicated in YAP1/TAZ signaling and the junctional morphology observed here is consistent with EC with YAP1/TAZ deficiency (Neto et al. 2018), we hypothesized that XAV939 treatment would impair YAP1/TAZ signaling in EC. Indeed, a robust decrease in YAP1/TAZ protein levels was observed that was accompanied by attenuated TEAD-mediated transcription. The decrease in YAP1/TAZ activity is likely the consequence of increased levels of the YAP1/TAZ negative regulators AMOTL1/2, which is consistent with previously published work on tankyrase inhibition in other cell types (Wang et al. 2015, Troilo et al. 2016, Wang et al. 2016a, Jia et al. 2017, Dattani et al. 2022). Interestingly, although functional YAP1/TAZ is crucial for VEGF/VEGFR2 signaling (Wang et al. 2017), we observed that XAV939 treatment results in an increase in VEGFR2 protein levels, potentially due to transcriptional upregulation upon YAP1/TAZ depletion. However, this increase did not enhance VEGF signaling. Instead, the accumulated VEGFR2 protein appeared to be retained in the Golgi apparatus, suggesting that XAV939 inhibited the normal trafficking of VEGFR2 to the cell surface, which is consistent with earlier published observations (Wang et al. 2017). Furthermore, XAV939 treatment led to impaired sprouting angiogenesis and EC migration, characterized by reduced filopodia formation, decreased CDC42 activation, and suppression of JAIL formation. These effects overlap with the known effects of inhibiting YAP1/TAZ signaling (Cao et al. 2017, Kim et al. 2017, Sakabe et al. 2017), supporting the notion that XAV939 functions as a potent YAP1/TAZ inhibitor in EC. YAP1/TAZ and the actin cytoskeleton have a bidirectional interaction where different actin structures can either enhance or suppress YAP1/TAZ and vice versa. Here, we demonstrate that inhibiting YAP1 activity, whether by XAV939 or by YAP1 depletion, induces the formation of radial F-actin bundles, which was associated with the recruitment of vinculin to sites of junctional remodeling, giving rise to focal adherens junctions (Huveneers et al. 2012). These results are consistent with published observations demonstrating that YAP1 regulates expression of different RhoGAPs (e.g., ARHGAP29 (Qiao et al. 2017), DLC1 (van der Stoel et al. 2020)) and thereby regulates F-actin bundling. Indeed, overexpression of YAP1 resulted in decreased F-actin bundling, which was intriguingly accompanied by increased TAZ nuclear translocation. Similarly, inhibiting F-actin bundling using the ROCK inhibitor H1152 promotes nuclear translocation of TAZ. Whereas it is generally assumed that increased F-actin bundling increases YAP1/TAZ activity, we observed a clear decrease in YAP1/TAZ activity after XAV939 with increased F-actin bundling, and conversely increased TAZ nuclear translocation after decreased F-actin bundling using H1152. Although these results are seemingly contrasting the well-established model in which F-actin bundling promotes YAP1/TAZ activity, recently published work by others has provided a clear model in which F-actin bundling at AJs (as is the case with focal adherens junctions) has an opposite effect on YAP1/TAZ activity compared to F-actin bundling at focal adhesion sites (Furukawa et al. 2017). Therefore, it appears that the interplay between YAP1/TAZ and the actin cytoskeleton is more complex than previously assumed and tankyrase inhibition greatly affects this interplay. Although YAP1 and TAZ have historically been described to possess functional redundancy, we observe that overexpression of YAP1 results in a loss of F-actin bundling and concomitant nuclear TAZ localization, suggesting that YAP1 and TAZ are regulated independently in EC. Indeed, non-overlapping expression of YAP1 and TAZ has been observed before in the vasculature (Neto et al. 2018) and several studies support the notion that YAP1 and TAZ differ in their respective transcriptional (Zanconato et al. 2015) and cytoplasmic roles. It has been suggested that, by virtue of their cytoplasmic half-life, TAZ predominantly functions as a transcriptional co-factor whereas YAP1 also regulates the actin cytoskeleton by non-transcriptional mechanisms.

### Possible implications for further development of tankyrase inhibitors for clinical use

Taking our results into account for the further development of tankyrase inhibitors as candidate drugs for cancer therapy, it is crucial that future studies go beyond its use as a Wnt/β-catenin inhibitor and address this interplay in different cell types and organ systems. In the recently reported first-in-human study for stenoparib, escalating doses up to 800 mg/day have been used (Plummer et al. 2020). Effective antitumor activity was reached at 400–600 mg/day. Eight hundred milligrams per day was regarded as not tolerable by the patients. The maximal serum concentrations reached in the steady state amounted to 1100 (400 mg/day)–2200 (600 mg/day) ng/ml which is equivalent 3.5–7 µM. Taking the reported IC_50_ for tankyrase 1 and 2 inhibition, 50–100 nM, as well as the IC_50_ for XAV939 (10 nM) measured within the same assay into account (McGonigle et al. 2015), the standard dose of XAV939 to obtain in vitro antitumor activity (5 µM) used in our investigation would be definitely in the range to cause side effects based on endothelial malfunctions. Indeed, Plummer et al. (2020) reported side effects such as periorbital edema and maculopapular or erythematous rash occurring frequently at the effective doses. In general, on-target side effects of novel targeted therapeutics in cancer therapy are to be expected since the majority of these novel modalities are not specific for cancer-related biological processes but affect also other physiological or pathological processes (Timar and Uhlyarik 2022). For example, as we found VEGFR2 signaling to be impaired by XAV939 treatment, side effects observed by anti-VEGF therapy, such as bleeding, thromboembolic events, and wound healing disturbances (Timar and Uhlyarik 2022), might occur with tankyrase inhibitors as well. In addition, the potential therapeutic implications of tankyrase inhibitors are not limited to oncology but has also disseminated to multiple sclerosis (Fancy et al. 2011), pulmonary fibrosis (Ulsamer et al. 2012), and lipid disorder (Wang et al. 2020), highlighting the need for minimizing off-target effects and toxicity.

### Use of ROCK inhibitors as counter measure to minimize endothelial side effects of tankyrase inhibition

The ROCK inhibitor fasudil is currently approved for the treatment of cerebral vasospasm in Japan and China, and reported to be efficacious in the treatment of pulmonary arterial hypertension (PAH) in patients (Zhang and Wu 2017). Based on our results, it is therefore tempting to speculate that the use of ROCK inhibitors, such as fasudil, in conjunction with tankyrase inhibitors might prove a potential strategy to limit at least the endothelial dysfunction. As inhibition of Wnt/$\beta$-catenin and thus tankyrase inhibition have been implicated in the treatment of PAH (de Jesus Perez et al. 2014), such patients might even benefit from the combination. Nevertheless, although apparently tolerable, side effects of a systemic application of ROCK inhibitors have to be considered. For example, in animal models an undesirable drop in blood pressure and a reduction in the lymphocyte count have been described (Kast et al. 2007, Shaw et al. 2014). Therefore, the outcome of such combinations is hardly predictable and has to be thoroughly validated before considered for the treatment of patients.

### Study limitations

XAV939 is the prototypical tankyrase inhibitor and was used at the same concentration generally admitted in cell culture experiments to achieve its inhibitory activity on cancer cells. Nevertheless, although the mode of action is similar, whether our results can be extended to other tankyrase inhibitors, such as stenoparib, has not been part of our investigation. Moreover, all results reported herein were obtained from an established EC culture and the ex vivo angiogenesis model of explanted p5 mouse retinas. Therefore, a proof of concept in a relevant animal model was also not part of this study. The same limitations apply to the use of the ROCK inhibitor H1152 to antagonize the effects of XAV939 treatment on the endothelium.

## Conclusions

Our study clearly revealed that alterations in other signaling pathways than the canonical Wnt/β-catenin signaling are relevant for the effects of tankyrase inhibitors on the endothelium. The upregulation of AMOTL1/2 induces profound alterations in the localization of the Hippo pathway mediators YAP1 and TAZ with profound consequences on gene transcription, AJ dynamics, endothelial permeability, and angiogenic behavior. Obviously, at least in EC, YAP1 and TAZ have distinct functions based on their interactions with and regulation of the actin cytoskeleton. The proposed model to explain these observations however requires further proof, e.g., in relevant animal models, in which also the differences between YAP1- and TAZ-mediated signaling can be addressed.

## Data Availability

The datasets generated during and/or analyzed during the current study are available from the corresponding author on reasonable request.

## Abbreviations

ADP: Adenosine diphosphate
AJ: Adherens junction
AMOTL1: Angiomotin-like 1
AMOTL2: Angiomotin-like 2
ANKDR1: Ankyrin repeat domain-containing protein 1
CDC42: Cell division control protein 42 homolog
EC: Endothelial cell
FITC: Fluorescein isothiocyanate
FOXO3a: Forkhead box O3a
GFP: Green fluorescent protein
GTP: Guanosine triphosphate
H1152: (S)-(+)-2-Methyl-1-[(4-methyl-5-isoquinolinyl)sulfonyl]-hexahydro-1H-1,4-diazepine dihydrochloride
HOPFlash: Luciferase reporter gene assay for TEAD-mediated transcription
HUVEC: Human umbilical cord endothelial cell
IF: Immunofluorescence
IP: Immunoprecipitation
JAIL: Junction-associated intermittent lamellipodia
OSS: Oscillatory shear stress
RhoGAP: GTPase activating protein for Rho monomeric GTPases
ROCK: Rho-associated protein kinase
TAZ: Transcriptional co-activator with PDZ-binding motif
TCF/Lef: T cell factor/lymphoid enhancer factor family of transcription factors
TEAD: TEA domain family of transcription factors
TOPFlash: Luciferase reporter gene assay for TCF/Lef-mediated transcription
TRITC: Tetramethylrhodamine isothiocyanate
VE-cadherin: Vascular endothelial-cadherin (CDH5)
VEGF: Vascular endothelial growth factor
VEGFR2: Vascular endothelial growth factor receptor 2
WB: Western blot
YAP1: Yes-associated protein 1
XAV939: 3,5,7,8-Tetrahydro-2-[4-(trifluoromethyl)phenyl]-4H-thiopyrano[4,3-d]pyrimidin-4-one
